# Strategies for Reducing Toxicity and Enhancing Efficacy of Chimeric Antigen Receptor T Cell Therapy in Hematological Malignancies

**DOI:** 10.3390/ijms24119115

**Published:** 2023-05-23

**Authors:** Haobing Wang, Ling Tang, Yingjie Kong, Wen Liu, Xiaojian Zhu, Yong You

**Affiliations:** 1 Institute of Hematology, Union Hospital, Tongji Medical College, Huazhong University of Science and Technology, Wuhan 430022, China; 2 Department of Pain Treatment, Union Hospital, Tongji Medical College, Huazhong University of Science and Technology, Wuhan 430022, China; 3 Department of Hematology, Tongji Hospital, Tongji Medical College, Huazhong University of Science and Technology, Wuhan 430030, China

**Keywords:** hematological malignancies, CAR-T therapy, toxicity, tumor progression, efficacy

## Abstract

Chimeric antigen receptor T cell (CAR-T) therapy in hematologic malignancies has made great progress, but there are still some problems. First, T cells from tumor patients show an exhaustion phenotype; thus, the persistence and function of the CAR-Ts are poor, and achieving a satisfactory curative effect is difficult. Second, some patients initially respond well but quickly develop antigen-negative tumor recurrence. Thirdly, CAR-T treatment is not effective in some patients and is accompanied by severe side effects, such as cytokine release syndrome (CRS) and neurotoxicity. The solution to these problems is to reduce the toxicity and enhance the efficacy of CAR-T therapy. In this paper, we describe various strategies for reducing the toxicity and enhancing the efficacy of CAR-T therapy in hematological malignancies. In the first section, strategies for modifying CAR-Ts using gene-editing technologies or combining them with other anti-tumor drugs to enhance the efficacy of CAR-T therapy are introduced. The second section describes some methods in which the design and construction of CAR-Ts differ from the conventional process. The aim of these methods is to enhance the anti-tumor activity of CAR-Ts and prevent tumor recurrence. The third section describes modifying the CAR structure or installing safety switches to radically reduce CAR-T toxicity or regulating inflammatory cytokines to control the symptoms of CAR-T-associated toxicity. Together, the knowledge summarized herein will aid in designing better-suited and safer CAR-T treatment strategies.

## 1. Introduction

Cellular adoptive immunotherapy is a treatment in which immunoreactive cells that have been activated and amplified in vitro are injected into patients. Nonspecific cellular adoptive therapies include lymphokine-activated killer cells (LAKs) and cytokine-induced killers (CIKs). Specific cellular adoptive therapies include tumor-infiltrating lymphocytes (TILs), TCR-T therapy, and CAR-T therapy. CAR-T therapy has a significant advantage in hematological malignancies and has been widely studied. However, CAR-T therapy also has certain problems that remain to be solved, such as poor efficacy and persistence in some patients, obvious CAR-T-associated toxicity, tumor recurrence, and other problems [1,2]. To reduce its toxicity and enhance its efficacy, researchers are using gene-editing technologies or anti-tumor drugs to explore new strategies throughout the process of designing, manufacturing, and using CAR-Ts.

For example, T cells were gene-edited to knock out the *NO4A* and *TOX* genes and overexpress argininosuccinate synthetase (ASS) before CAR lentivirus transfection, thus improving CAR-T function [3,4,5]. Strategies such as the inhibition of the PI3K signaling pathway and the BET protein in T cells [6,7] induce a better functional status and differentiation phenotype of T cells. When designing CAR structures, tandem CARs, dual CARs, and tri-specific CARs are constructed, which enable CAR-Ts to respond to multiple antigens, enhance their efficacy, and prevent antigen-negative tumor recurrence [8,9,10]. Researchers installed a “safety switch” in the CAR structure which enabled medical personnel to control the activity of CAR-Ts or completely eliminate CAR-Ts when serious toxicity occurred [11,12,13,14,15,16,17]. There were also studies that used other anti-tumor drugs, such as BTK inhibitors and immune checkpoint inhibitors, which can enhance the efficacy and activity of CAR-Ts. [18,19,20]. In addition, anti-inflammatory cytokine drugs such as the IL-6R monoclonal antibody tocilizumab, IL-1R monoclonal antibody anakinra, and a GM-CSF antibody were used to reduce the toxicity of CAR-T therapy [21,22,23]. These strategies have shown satisfactory effects in enhancing the efficacy and reducing the toxicity of CAR-T therapy. Understanding them will aid medical personnel in better using CAR-T therapies to cure hematological malignancies. The strategies for using the gene-editing technologies involved in this paper are described in Table 1. The strategies for using the drugs involved in this paper are described in Table 2.

## 2. Combining CAR-T Therapy with Gene Editing or Drug Assistance to Enhance CAR-T Persistence and Anti-Tumor Efficacy

In the field of gene editing, zinc finger nucleases, TALEN, and CRISPR-cas9 have been used to edit the genes of cells. Researchers hope to use these strategies to enhance the activity of CAR-Ts or to re-engineer CAR-Ts into universal CAR-Ts, known as off-the-shelf CAR-Ts, to reduce the risk of host versus graft disease [102,103]. Zinc finger nucleases (ZFN) consists of a zinc finger protein (ZFP) and a Fok I endonuclease, which can recognize and spot-cut DNA to achieve gene-editing purposes. Researchers usually use plasmid transfection to produce a zinc finger nuclease in the cell for this function [104]. Lipid nanoparticles (LNPs) are also used to deliver zinc finger nuclease mRNA into cells for gene editing [105]. TALEN is composed of a transcription-activator-like (TAL) effector and nuclease, which recognize and bind specific DNA sequences. It has the same gene-editing effect as ZFN. Electroporation is commonly used to deliver TALEN mRNA into cells. In the field of CAR-T therapy, the presence of TCR on the surfaces of CAR-Ts can lead to host versus graft disease, which makes constructed CAR-Ts available only to the donor. The TALEN technique was used to knock out the TCR of CAR-Ts and successfully construct a universal CAR-T, reducing the risk of host anti-graft disease [106]. The CRISPR/Cas9 system consists of two parts: the Cas9 protein, which performs DNA cutting, and the sgRNA, which has a guiding function. The RNP method, viral transfection, plasmid transfection, and the transposon method were used to transfer gRNA and Cas9 proteins into cells to complete gene editing. In the field of CAR-T therapy, researchers have demonstrated that this system can effectively perform gene editing on T cells and CAR-Ts [107,108]. Recently, researchers have developed a new gene-editing technique called CRISPR-based library-scale AAV perturbation with simultaneous HDR knock-in (CLASH), which can achieve unitary and rapid transgene knock-in [109].

### 2.1. Pretreating CAR-Ts to Enhance Their Activity

Orphan nuclear receptor NR4A protein is associated with the exhaustion of CD8+ T cells [24]. In terms of the mechanism, NR4A can promote the acetylation of histone 3 at lysine 27 (H3K27ac), leading to the activation of immunotoleration-related genes. NR4A can also inhibit AP-1, thus inhibiting the expression of T cells’ effector genes. CD8+ T cells from tumor patients express high levels of NR4A transcription factors, thus limiting the function of CAR-Ts. *NR4A*-knockout CAR-Ts enhanced anti-tumor function, promoted tumor reduction in vitro [4,25].

Another gene that can be targeted to enhance CAR-T efficacy is *TOX*. The DNA-binding protein TOX can cause T cell exhaustion. *TOX* knockout enhances CAR-Ts’ persistence and tumor-killing activity [26,27]. This may be due to the reduced expression of T cells’ surface inhibitory receptors, such as Pdcd1, Entpd1, Havcr2, Cd244, and Tigit, via *Tox* knockout [5]. In addition, Regnase-1-deficient CD8+ T cells expressed higher levels of memory-T-cell-associated genes and lower levels of TOX expression. These cells significantly increased anti-leukemia activity and persistence in a mouse model [28].

In addition to gene editing, using drugs to affect the functions of proteins can also have an effect. PI3K inhibitors can regulate the differentiation of CAR-Ts to maintain a lower differentiation phenotype, improve in vivo persistence, and reduce the tumor burden in mice models [7]. Dual PI3Kδ/γ inhibition during CAR-Ts’ expansion procedure maximized the number of central memory T stem cells and naive T cells and decreased the expression of the TIM-3 exhaustion marker. CAR-Ts treated with duvelisib increased the expression of mitochondrial fusion protein MFN2, thus increasing the relative mitochondrial content. These CAR-Ts showed a significant increase in cytotoxicity against CD8+ chronic lymphocytic leukemia (CLL) target cells in vitro. In a mouse model, these CAR-Ts expanded significantly faster, persisted longer, and eliminated tumors more quickly [59].

Another protein that can be targeted is BET. Bromodomain and extra-terminal motif (BET) protein BRD4 directly regulate the expression of transcription factor BATF in CD8+ T cells, which can promote the differentiation of CD8+ T cells into effector T cells. JQ1, an inhibitor BET protein, enables CD8+ T cells to exhibit stem-like or central memory T cell characteristics by inhibiting the expression of the transcription factor BATF. JQ1-treated CAR-Ts showed enhanced persistence and anti-tumor effects in vitro. In addition, researchers found that histone acetyltransferase p300 can also promote *BATF* transcription, and the inhibition of p300 enhanced the anti-tumor effect of adoptive transferred T cells. These results suggest that targeting BATF signals can generate superior CAR-Ts for clinical purposes [6].

In addition to transcriptional factors, proteins that exert enzymatic activity can also affect cell activity. Tumor cells consume large amounts of arginine to drive cell proliferation, and the resulting low-arginine microenvironment impairs the proliferation of CAR-Ts. In addition, the expression of argininosuccinate synthetase (ASS) and ornithine transcarbamylase (OTC), which are involved in arginine synthesis, was low in T cells. Gene editing T cells to express functional ASS or OTC enzymes can promote the proliferation of CAR-Ts without increasing the T cells’ exhaustion. These modified CAR-Ts effectively mediated tumor clearance in vivo [3].

### 2.2. Combining CAR-Ts with Other Anti-Tumor Strategies

In addition to cell therapy, immunotherapy includes immune checkpoint inhibitors, anti-cytokine antibodies, and chemotherapy drugs. Here, we will explore how these drugs can be combined with CAR-T therapy to enhance its efficacy.

Ibrutinib enhances the activity of CAR-Ts and promotes their expansion by down-regulating the programmed cell death protein 1 (PD-1) on the T cells‘ surfaces. It also down-regulates CD200, an immunosuppressive molecule, on tumor cells. In lymphocytic leukemia xenotransplantation models, ibrutinib improved CAR-T implantation and tumor clearance [18,60,61]. The addition of ibrutinib when manufacturing CAR-Ts increased the activity and expansion capacity of the CAR-Ts. The resulting CAR-Ts enriched the phenotypes of less-differentiated naive cells and decreased the expression of exhaustion markers such as PD-1/TIM-3/LAG-3 [62]. Ibrutinib also reduces serum cytokine levels during CAR-T therapy and prevents the occurrence of cytokine release syndrome (CRS) [63].

BCL-2 inhibitors can inhibit the binding of BCL-2 and BAX proteins to form a dimer so that the tumor cells can re-establish their normal apoptosis capacity. It has now been shown that the pre-sensitization of B-acute lymphoblastic leukemia (B-ALL) tumor cells with the BCL-2 inhibitor venetoclax can up-regulate the expression of CD19 and pro-apoptotic proteins on tumor cells. The subsequent use of CAR-T therapy can achieve a satisfactory killing effect and maintain CAR-Ts [64].

Tumor cells’ expression of indoleamine 2,3-dioxygenase (IDO) can convert tryptophan into metabolites that inhibit central immune function [65]. In the field of CAR-T therapy, tryptophan metabolites inhibit cytokine-dependent amplification, cytotoxicity, and cytokine secretion with CAR-Ts. CD19 CAR-Ts could not function on IDO-positive tumors. IDO inhibitors (1-methyltryptophan) improved the anti-tumor efficacy of CD19 CAR-Ts in lymphoma. In addition, fludarabine and cyclophosphamide, which were routinely used before CAR-T therapy, down-regulated IDO expression in lymphoma cells and improved the anti-tumor activity of CD19-CAR-T in vivo [66].

Exhausted T cells are present in tumor patients and are described as a subpopulation of T cells with significant losses of proliferative potential and effector function. These exhausted T cells also consistently overexpress multiple immune checkpoint receptors such as PD-1/TIM-3/LAG-3/CTLA-4 [67,68]. Studies have shown that the activity of CAR-Ts constructed from these exhausted T cells is significantly limited, ultimately affecting the efficacy and outcome of CAR-T therapy. That is, exhausted T cells are associated with poor molecular responses [69,70]. In a clinical trial of CD19 CAR-Ts, the no response (NR) group showed a higher frequency of exhausted T cells. High frequencies of TIM3+ and LAG3+ T cells upon cell collection predicted the failure of CAR-T therapy [71]. Worse, in AML/CML/ALL, PD-L1 was abnormally expressed on tumor cells, which combined with PD-1 on the surface of the exhausted T cells to further impair CAR-T activity [19,72,73].

In preclinical trials, blocking PD-1/PD-L1 interactions through the use of PD-L1 antibodies in a mouse model restored the function of T cells function and prolonged mouse survival [19]. Using gene-editing technology to knock out the *PD-1* gene in T cells can also achieve a similar effect. [74,75,76]. In CLL, the use of antibodies to block LAG-3 improved the activation of T cells [20]. Additionally, blocking CTLA-4 in combination with CAR-T therapy has also been shown to be beneficial [77].

In clinical trials, the PD-1 inhibitor pembrolizumab was effective and safe for CD19 CAR-T therapy in patients with relapsed B-ALL, enhancing the effects and persistence of the CAR-T [72]. In a clinical trial of 11 non-Hodgkin lymphoma (NHL) patients, CD19 CAR-T combined with nivolumab (PD-1 inhibitor) mediated severe anti-lymphoma activity. The objective response rate (ORR) and complete response (CR) rate were 81.81% (9/11) and 45.45% (5/11), respectively. In addition to the powerful curative effect, all the side effects were controllable and reversible. Overall, the combination of immunocheckpoint inhibitors and CAR-T therapy showed good efficacy and safety [78].

## 3. Enhancing the Anti-Tumor Efficacy of CAR-Ts and Preventing Tumor Recurrence in the Process of Their Design, Manufacture, and Usage

### 3.1. Adjusting the Design of CAR Structure

Conventional CAR structures recognize single-tumor-specific antigens, such as CD19 and CD20, and mediate T cell effects. However, not all tumor cells express what we identify as tumor-specific antigens. For example, the presence of CD19-negative tumor cells in B-ALL patients before anti-CD19 CAR-T therapy leads to antigen-negative tumor recurrence [110]. In addition, the survival stress induced by CAR-T therapy also induces acquired mutations in B-ALL tumor cells, resulting in tumor cells with a CD19-negative phenotype. These CD19-negative tumor cells can escape the killing of CD19 CAR-Ts [111]. Researchers have constructed novel CAR structures that can simultaneously recognize two or more antigens, thus solving this problem.

**Figure 1:**
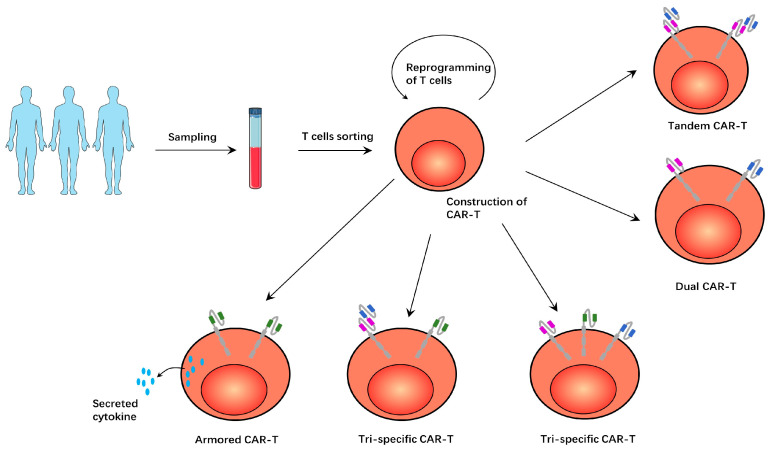
T cells obtained from patients were modified to construct various CAR-Ts. In tandem CAR-T, T cells express a CAR structure with two antigen-binding domains. In dual CAR-T, T cells expressed two CAR structures with an antigen-binding domain on each CAR structure. In tri-specific CAR-T, T cells express three CAR structures, each of which has an antigen-binding domain. In another tri-specific CAR-T, T cells express two CAR structures, a tandem CAR structure and another conventional, single-tumor-specific CAR structure. In armored CAR-T, the T cells secrete cytokines that promote the immune response, enhancing the anti-tumor effect.

Researchers have constructed tandem CAR structures with bispecific properties. The antigen-binding domain of this CAR structure has two tandem scFvs that recognize both CD19 and CD20. This tandem CAR-T structure is shown in Figure 1. Researchers demonstrated that such a bispecific antigen recognition domain does not affect the growth, differentiation, and lytic ability of CAR-Ts. Such tandem CAR-Ts can identify CD20 in the absence of CD19 molecules in tumor cells, thus solving the problem of antigen-negative tumor recurrence [9,49]. In preclinical trials, CD19/CD22 tandem CAR-T produced interferon-γ(IFN-γ) and interleukin-12 (IL-12) equivalent to monospecific CAR-Ts and eradicated tumor-cell-line xenografts and patient-derived tumor xenografts (PDX) [50]. In addition, both CD19/CD79b and CD19/CD37 tandem CAR-T treatments showed efficacy in preclinical trials [51,52]. In clinical trials, CD19/CD22 tandem CAR-Ts demonstrated safety and significant anti-leukemia activity in refractory/relapsed B-ALL patients [53,54]. CD19/CD20 tandem CAR-T treatment elicits an effective and durable anti-tumor response in refractory/relapsed NHL patients, with a complete response rate (CRR) of 71% and a progression-free survival rate at 12 months of 64% [55]. In addition to avoiding antigen-negative tumor recurrence, tandem CAR-Ts were less toxic with a higher disease burden, possibly due to their optimized tumor-killing activity and moderate cytokine production [56].

In addition to tandem CAR-T, the simultaneous expression of two CAR structures on dual CAR-Ts had the same effect. This dual CAR-T structure is shown in Figure 1. The researchers found that CD123 was up-regulated in leukemia-initiating cells and CD19-negative blast cells in B-CLL. They therefore used two lentiviruses to construct dual CAR-Ts expressing both CD19 CAR and CD123 CAR structures. In in vivo and in vitro studies, these dual CAR-Ts demonstrated enhanced anti-tumor activity when compared to monospecific CAR-Ts and certainly overcame the problem of CD19-negative recurrence in B-ALL [8]. In clinical trials, dual CAR-Ts expressing both CD19 CAR and CD22 CAR demonstrated safety and efficacy in pediatric/young adult B-ALL patients [57].

There are even CAR-Ts that express three antigen-specific CAR structures at the same T cell, which are called tri-specific CAR-Ts. These CD19/20/22 tri-specific CAR-Ts were effective at killing tumor cells in vitro and in vivo and were effective at preventing antigen-negative tumor recurrence. Tumor cells expressing any of these three antigens will be recognized and killed by the tri-specific CAR-Ts [58]. This CAR-T structure is shown in Figure 1. There is another tri-specific CAR-T that combines tandem-CAR and dual-CAR features to express a conventional CD22 CAR and a tandem CD19/CD20 CAR. These tri-specific CAR-Ts showed enhanced cytolytic activity in vitro and were effective against CD19-negative target cells. In animal models, when monospecific CAR-T is not effective, this tri-specific CAR-T can still effectively kill tumors. [10]. This tri-specific CAR-T structure is shown in Figure 1.

In addition to modifying CAR structures to achieve better tumor-killing efficacy, gene editing CAR-Ts to secrete cytokines that promote the immune response is also a strategy. This CAR-T is called the fourth generation CAR-T or armored-CART. IL-12 can stimulate T cells to produce interferon-γ (IFN-γ), induce the apoptosis of Treg, and increase the infiltration of CD8+ T cells to enhance the immune response [112,113]. In addition, IL-12 can also increase the expression of CD80, CD86, OX-40L, and other co-stimulatory molecules on the surface of myeloid-derived suppressor cells (MDSCs), weaken the T-cell-inhibiting function of MDSCs, and enhance the anti-tumor immune response [114]. CAR-Ts engineered to express IL-12 showed enhanced lethal activity in lymphoma models. These IL-12-secreting CAR-Ts not only kill tumor cells better but also recruit immune cells to perform an anti-tumor immune response [115]. IL-18-secreting CAR-Ts showed enhanced in vivo expansion and persistence and significantly improved long-term survival in hematologic tumor mouse models. In addition, the IL-18 secreted by CAR-Ts modulates the tumor microenvironment and induces the amplification of immunoeffector cells such as natural killer cells, NKT cells, dendritic cells (DC), and CD8+ T cells, thereby enhancing the endogenous anti-tumor immune response [116]. Compared with conventional CD19 CAR-Ts, IL-15-secreting CD19 CAR-Ts showed greater amplification, lower cell mortality, decreased expression of PD-1, and an enhanced anti-tumor effect in vivo [117]. This CAR-T structure is shown in Figure 1.

### 3.2. Optimizing the Manufacturing Process of CAR-Ts

The exhaustion and senescence of T cells affect their activity and function. T cells in patients with hematological malignancies exhibit an exhaustion phenotype, and CAR-Ts constructed from autologous T cells of these patients also exhibit an exhaustion phenotype [118]. CD4+T cells in CLL patients expressed high levels of exhaustion markers such as PD-1, CD160, and CD244. CD8+ T cells showed a decrease in their proliferative capacity and cell activity [119]. T cells from CLL patients had a lower proportion of naive T cells than T cells from normal subjects, and CAR-Ts constructed from such T cells also had a poor expansion potential and poor long-term maintenance capacity [120,121]. Some studies have reprogrammed antigen-specific CD8+ T cells into induced pluripotent stem cells (IPSCs) and induced them to re-differentiate. These re-differentiated T cells have a high proliferative capacity and long telomeres and have an enhanced antigen-specific killing activity [122,123]. This method fundamentally reverses the exhaustion status of the T cells and restores their activity. CAR T cells constructed using such “enhanced” T cells should exhibit greater cell activities and a lower percentage proportion of exhausted cells.

The lentivirus infection temperature appears to influence the phenotype and function of the resulting CAR-Ts. The results showed that CAR-Ts produced via the lentivirus transfection of T cells at 32 degrees had the largest proportion of naive cells, the lowest immune checkpoint receptor expression, and the strongest tumor-cell-killing activity. These CAR-T seems to strike a balance between function and phenotype for optimal clinical outcomes [124].

IL-2 is routinely used in the process of T cell expansion in vitro, but high concentrations of IL-2 drive T cells to differentiate into effector cells and reduce the number of central memory T cells. Studies have shown that reducing the use of IL-2 and shortening the time of T cell amplification in vitro can increase the number of early memory T cells. The resulting CAR-Ts also showed better performance [125].

Studies have shown that lymphocyte depletion therapy prior to CAR-T infusion helps CAR-Ts function. One reason for this is that lymphocyte depletion therapy increases the levels of IL-7 and IL-15, which enhance the function and anti-tumor activity of T cells function [126]. Preclinical studies have shown that the addition of IL-7 and IL-15 in the process of the expansion of T cells can increase the number and proportion of CD8+CD45RA+CCR7+-naive T cells that are resistant to T-cell death induced by multiple exposures to antigens and thus exert a more powerful anti-tumor effect [127]. Other studies have shown that IL-15 enhances the anti-tumor activity of CAR-Ts and promotes the expansion of stem-cell-like memory subsets [128]. In addition, IL-15-treated CAR-Ts showed decreased expression of exhaustion markers, enhanced anti-apoptotic ability, increased proliferation ability, and improved mitochondrial activity [129]. rIL-21 can improve the activity and proliferation of T cells in aged mice and promote the development of new T cells [130,131]. The addition of IL-21 improved the function of CAR-Ts. Animal studies have shown that CAR-Ts cultured with IL-21 can effectively penetrate CD19+B cell tumor foci in mice [132].

### 3.3. Combining Multiple CAR-Ts to Increase Efficiency

Single-target CAR-T therapy sometimes fails to achieve a satisfactory response and is associated with antigen-negative tumor recurrence. The introduction of multiple CAR-Ts targeting several targets may solve this problem. There are three CD19-CART-resistant DLBCL patients who achieved complete responses (CRs) after treatment with CD22 CAR-Ts [133]. Another study evaluated sequential administration of CD19 and CD22 CAR-Ts in R/R B-ALL and NHL patients and concluded that this approach is safe and effective in mediating long-term remission in patients [134,135,136]. In addition, the sequential or simultaneous infusion of CD19/BCMA CAR-T and CD20/CD22 CAR-T was also studied [137,138,139]. There are even studies of the sequential infusion of three CAR-Ts. Researchers explored relapsed/refractory Burkitt lymphoma with the sequential infusion of CD19/CD22/CD20 CAR-Ts. Results show that this approach can induce lasting remissions in patients, and only a small number of patients develop severe CRS and neurotoxicity. This proves the safety and feasibility of this cocktail therapy [140,141].

## 4. Strategies to Reduce the Toxicity Related to CAR-T Therapy

### 4.1. Modifying the Structure of CAR to Alleviate Toxicity Fundamentally

The human immune recognition of murine-derived CAR structure may result in toxicity, especially after repeated injections of CAR-Ts, because single-chain antibody fragment (scFv) structures in murine-derived CAR structures are immunogenic. The use of human or humanized scFvs may reduce immunogenicity. Researchers used humanized scFvs to construct CD19 CAR-Ts that were activated by CD19+ tumor cells and successfully eliminated human lymphoma grafts in a mouse model. Such a CAR structure may reduce the immune rejection reaction and the allergic reactions associated with murine-derived scFvs, thereby reducing patients’ side effects [142,143]. Researchers evaluated the clinical efficacy of humanized CAR-T infusion in R/R ALL patients and showed that the transport ability and tumor cell lysis activity of humanized CAR-Ts were superior to those of murine CAR-T. In addition, humanized CAR-Ts’ immunogenicity decreased, and their persistence increased [144]. Others evaluated the safety and feasibility of humanized CD19 CAR-Ts in B-cell lymphoma. The results showed that humanized CAR-Ts had a similar anti-tumor activity to murine CAR-Ts. In addition, humanized CD19 CAR-Ts produced lower levels of cytokines, and the probability of severe neurotoxicity was significantly reduced from 50% to 5% [145]. Another study showed similar results, with humanized CAR-Ts showing significant lethal activity and lower neurotoxicity against lymphoma [146].

The hinge region and transmembrane region of the CAR structure can influence the expression level and stability of CARs [1]. The commonly used hinge regions include the amino acid sequences of CD8α, CD28, and IgG. However, CAR-Ts constructed using the IgG-derived hinge region showed a lack of anti-leukemia activity in vivo and induced severe cytotoxicity. It has been shown that the CH2CH3 domain derived from IgG binds to the Fcγ receptor and mediates the activation of T cells. This leads to the exhaustion of T cells and cytotoxicity [147]. Of course, the current results do not indicate that IgG should not be used as the hinge region of CAR-T. CAR-Ts using the hinge region derived from IgG in multiple studies also have shown activity and effectiveness [148,149,150,151]. In addition, we can induce mutations to eliminate the FCγ-receptor-binding ability of IgG [150,152]. CAR-Ts with a CD8α-derived hinge region and transmembrane region released fewer cytokines and showed a reduced susceptibility to activation-induced cell death when compared with CD28. The ability of the two different CAR-Ts to eliminate tumor cells was comparable. A preclinical study indicates that CAR-Ts with a CD8-derived transmembrane and hinge domain can increase persistence and decrease cytotoxicity without decreasing their anti-tumor activity [153,154]. In a clinical study of CAR-Ts using CD8 as hinge and transmembrane regions, 6 out of 11 B-cell lymphoma patients showed complete remission (CR), and no primary CRS or neurotoxicity were found in all 25 patients. In addition, no significant increase in serum cytokines was observed in all patients. CAR-Ts proliferated and differentiated into memory cells in vivo. These results confirmed the safety and efficacy of CAR-T with CD8 as hinge region and transmembrane region [155].

CD28 and 4-1BB were the most common costimulatory domains, and different costimulatory domains showed different effects on the differentiation of CAR-Ts. CAR-Ts containing the CD28 costimulatory domain were more likely to have all grades and severe CRS than the 4-1BB costimulatory domain [156]. Preclinical studies have shown that the CAR structure with the CD28 costimulatory domain differentiates primarily into effector T cells that utilize aerobic glycolysis for energy. However, CAR-Ts with a 4-1BB costimulatory domain mainly differentiated into central memory T cells, which showed increased mitochondrial biosynthesis and oxidative metabolism. In addition, the 4-1BB downstream signal activates NF-κB activity via TNFR-associated factors (TRAF), ultimately leading to the up-regulation of proteins involved in the regulation of cell cycle and survival, such as MYC, cyclin D1, BCL-XL, and BCL-2 [157]. Intuitively, the CD28 costimulatory domain was associated with a rapid initial expansion and the subsequent exhaustion and death of CAR-Ts, and the 4-1BB domain was associated with lower peak levels of T cell expansion, longer T cell persistence, and a lower risk of toxicity. Therefore, CAR-Ts with the 4-1BB costimulatory domain may be preferred for patients who are sensitive to CAR-T therapy [158,159,160,161,162]. Of course, CD28 should be selected for better anti-tumor activity if the patient has a lower tumor-specific-antigen density and is expected to tolerate cytotoxicity well [153].

### 4.2. Targeting Uncontrolled Inflammatory Responses

After making contact with tumor-cell-surface antigens, CAR-Ts activate and produce cytokines, including IFN-γ, TNF-α, and IL-2. The autologous immune cells bind to these cytokines and also release IL-10 and proinflammatory cytokines such as IL-1, IL-6, and IFN-γ. This is an important part of the immune response, but sometimes high levels of inflammatory cytokines will affect multiple organ functions, leading to cytokine release syndrome (CRS) and associated neurotoxicity [163].

IL-1 and IL-6 are mainly derived from human monocytes and are essential cytokines for CRS and neurotoxicity [164,165]. Elevated serum IL-6 levels were highly associated with severe CRS and neurotoxicity [166,167]. IL-6 can regulate the proliferation and differentiation of immune cells through membrane-binding IL-6 receptor (mIL-6R) and soluble IL-6 receptor (sIL-6R) [168,169]. IL-6 was also shown to enhance the proliferation and anti-tumor activity of CAR-Ts [121,170]. Tozzizumab is a humanized monoclonal antibody against the IL-6 receptor (IL-6R) that has been used to treat arthritis and Crohn’s disease [79]. Researchers used tocilizumab to target IL-6R to control cytokine release syndrome (CRS) without affecting the proliferation and anti-tumor activity of CAR-Ts, alleviating clinical symptoms [80,81,82,83]. These two points appear to be contradictory, possibly because although tocilizumab blocks IL-6R, IL-6R-mediated IL-6 consumption is reduced, ultimately leading to an increase in serum IL-6 levels [84]. The effect of IL-6 receptor blocking and elevated IL-6 levels on CAR-Ts appeared to be neutralized, and therefore the activity of CAR-Ts was not affected. Additionally, tocilizumab was approved by the FDA in 2017 for the treatment of CAR-T-associated CRS in adults and children over 2 years of age [21]. However, because tocilizumab increases serum IL-6 levels and cannot enter the central nervous system, it cannot control and may even aggravate neurotoxicity. Now, researchers are considering another IL-6 antibody. Siltuximab directly binds to the IL-6 molecule and prevents it from binding to soluble or membrane IL-6R, thereby inhibiting IL-6 activity [85,86]. Siltuximab successfully treated CRS in one patient, but more studies are needed in the CAR-T area [87]. In addition to antibody therapy, a non-signaling membrane-bound IL-6 receptor (mbaIL-6) has been designed. This mbaIL6 expressed on the surface of T cells can rapidly remove IL-6 from culture supernatant and neutralize IL-6 signaling transduction. CAR-Ts with mbaIL6 showed the same cytotoxicity and proliferative activity as cells expressing a CAR alone [88].

The IL-1 receptor antagonist anakinra has cerebrospinal fluid permeability and can relieve the central nervous system toxicity of patients and reduce the levels of IL-6 and TNF in the serum and cerebrospinal fluid of patients [89]. In studies, anakinra eliminated CRS and neurotoxicity and significantly extended leukemia-free survival [22,171]. In addition, anakinra can also treat hemophagocytic histiocytosis (HLA) [90].

In addition, IL-1 and IL-6 can induce the synthesis of inducible nitric oxide synthase (INOS), prompting macrophages to produce excess nitric oxide (NO), leading to the clinical manifestations of cytokine release syndrome with hypotension and increased vasodilation. The inducible nitric oxide synthase inhibitors L-NIL and 1400 W can reduce mortality and toxicity in mice [22].

GM-CSF is a CAR-T-derived key CRS-promoting protein [91]. High levels of serum GM-CSF were also associated with severe neurotoxicity [92,93]. A clinical trial of CAR-Ts in B-cell lymphoma showed that GM-CSF was significantly associated with the development of grade 3 or higher neurotoxicity [94]. Researchers demonstrated that the antibody-mediated neutralization or gene-editing technology-mediated inactivation of GM-CSF in CAR-Ts can eliminate macrophage-secreted CRS biomarkers such as MCP-1, IL-6, and IL-8, significantly reducing neurotoxicity and CRS occurrence. In addition, the anti-tumor function and proliferative capacity of CAR-Ts were not impaired in vitro [23,91,95]. In addition, GM-CSF promoted the amplification of MDSCs and increased the expression of PD-L1 on MDSCs, which inhibited the activity of CAR-Ts. This pathway was inhibited via GM-CSF neutralization therapy to restore the efficacy of CAR-Ts [96].

A TNF-α inhibitor (Etanercept) successfully cured CRS in two patients without altering the patients’ responses to CAR-T therapy, and no adverse events directly related to the administration of Etanercept were observed. In addition, Etanercept did not affect the proliferation and effector function of CAR-Ts in vitro [172,173]. However, the number of cases in this study was too few, and further research is needed.

Catecholamine, produced by macrophages, surges in CRS, driving the inflammatory response. Inhibiting catecholamine synthesis protected mice from lethal CRS. The administration of the catecholamine inhibitors methyltyrosine (MTP) or atrial natriuretic peptide (ANP) prior to CD19-CAR T cell therapy reduced plasma epinephrine and norepinephrine levels, as well as the plasma levels of IFN-α, TNF-α, and IL-6, and ultimately improved mouse survival without diminishing the expansion and tumor clearance of CAR-Ts [97].

The JAK-STAT pathway is a major signaling mechanism for a variety of cytokines. Itacitinib is a highly selective JAK-1 inhibitor that can interfere with cell signaling and affect the role of inflammatory cytokines. Itacitinib has been shown to significantly and dose-dependently reduce the levels of various cytokines associated with CRS in in vitro and in vivo models without significantly inhibiting the proliferation and anti-tumor activity of multiple CAR-Ts (CD19/EGFR/GD2) [98]. Additionally, studies have shown that the JAK inhibitor ruxolitinib is effective in the treatment of CRS and secondary hemophagocytic lymphohistiocytosis and has no significant effect on the proliferation and anti-leukemia effect of CAR-Ts [99,100].

### 4.3. Inhibiting CAR-T Activity Reversibly

Dasatinib, a tyrosine kinase inhibitor, interferes with lymphocyte-specific protein tyrosine kinase (LCK) in a dose-dependent manner by inhibiting CD3ζ and ZAP70ζ phosphorylation and deactivating CD3-based signaling transduction in the CAR structure. This induces CAR-Ts into a state of temporary inactivation. In in vivo and in vitro research studies, dasatinib inhibited the cytolytic activity of CAR-Ts and terminated cell proliferation and cytokine production. After the discontinuation of dasatinib, the inhibition was quickly and completely reversed, and the anti-tumor function of the CAR-Ts was restored [101]. In a mouse model of CRS, research studies demonstrated that the early administration of Dasatinib after CAR-T infusion can reduce the levels of Ifn-γ, Tnf-α, Gm-csf, and Il-2 in mice and significantly reduce the mortality of mice. Dasatinib inhibits CAR-Ts’ activity faster than dexamethasone and is expected to control neurotoxicity due to its blood–brain barrier permeability [12]. In this way, Dasatinib acts as a safety switch. We can use dasatinib to temporarily control CAR-Ts during severe toxicity and discontinue it when toxicity is reduced, thus restarting the CAR-T anti-tumor response.

**Figure 2:**
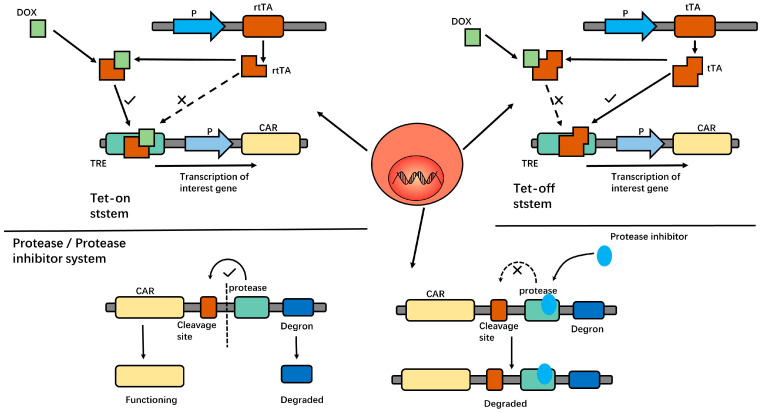
In the Tet-on system, the reverse tetracycline-controlled transactivator protein (rtTA) cannot bind to the Tet response element (TRE) to initiate downstream gene transcription in the absence of Dox. After DOX administration, the DOX—rtTA conjugate products can bind to TRE and thus initiate gene expression. In the tet-off system, tetracycline transcriptional activator (tTA) can directly bind to TRE to initiate gene expression under physiological conditions. After DOX administration, the DOX—tTA conjugate products cannot bind to TRE, thus inhibiting gene expression. In the protease/protease inhibitor system, under physiological conditions, the protease cleaves the entire structure from the cleavage site to release the CAR structure, and CAR-Ts function normally. After protease inhibitor administration, protease has no effect. Degron mediates the degradation of the entire structure, and the CAR structure could not be expressed on T cells. Thus, it can control the activity of CAR-Ts.

In another study, an inducible CD19 CAR-T (iCD19 CAR-T) was developed by integrating the Tetracycline transcription regulatory system (Tet-on) into the construction of the CAR structure. iCD19 CAR-Ts exhibited doxycycline-dependent cell proliferation, cytokine production, CAR expression, and antigen-specific cytotoxicity. After the removal of doxycycline, the CAR expression on T cells decreased significantly. These results suggest that the Tet-on system can control the activity of CAR-Ts, enhancing the safety of CAR-T treatment while maintaining its anti-tumor effect [41,42]. Similar results have been obtained in CD38 CAR-Ts [43]. In addition, the tet-off system was studied in CD5 CAR-Ts. In this system, the presence of doxycycline inhibits the CAR expression on T cells. The removal of doxycycline restored CAR expression and anti-tumor activity [44]. In this way, we can control the activity of CAR-Ts that are injected into patients. This tet-on/off system is shown in Figure 2.

Researchers have constructed a special protein structure which consists of a CAR, protease cleavage site, protease, and degron series. In the absence of a protease inhibitor, the protease cleaves the cleavage site, releasing the CAR construct, while the degron-bound-protease is targeted to the proteasome and thus degraded. In the presence of protease inhibitor Asunaprevir (ASN), the degron is not cleaved from the CAR, dragging the whole molecule to degradation [17]. Thus, CAR activity is controlled by this protease/protease inhibitor system. This protease/protease inhibitor system is shown in Figure 2.

Another reversible way to control CAR-Ts is to introduce a switching molecule between the tumor cells and CAR-Ts. The switching molecule consists of two components, a CAR-T binding domain and a tumor-specific antigen-binding domain. In the case of CD19+ tumor cells and anti-FITC CAR-T, the switching molecule consists of two parts: FITC and CD19 antibodies. Such switching molecules can link tumor cells to CAR-Ts. After CAR-Ts are injected into patients, the introduction of this switching molecule produces anti-tumor activity in a dose-dependent manner. In addition, the toxicity of CAR-T therapy can be controlled by changing the dosage of this switching molecule [11]. In addition to reducing toxicity, this particular structure has another advantage. After a single infusion of CAR-Ts, we can use several different switching molecules containing different types of antigen-binding domains to simultaneously target multiple tumor-specific antigens. This not only enhances the anti-tumor effect but also prevents antigen-negative recurrence caused by reduced expression of a single antigen on tumor cells [45]. Several similar studies have demonstrated the feasibility this approach [46,47,48].

The purpose of the above studies is to control CAR-Ts that can exist in the human body for a long time. However, mRNA CAR-T fundamentally avoids the long-term existence of CAR-T; here, the CAR-Ts die out naturally after exerting anti-tumor effects. In such a study, the T cells are briefly reprogrammed with mRNA to express the chimeric antigen receptor against tumor cells. The toxicity of these CAR-Ts was significantly reduced over time due to the unstable nature of the mRNA [174]. Multiple studies have investigated the efficacy of human T lymphocytes transfected with mRNAs encoding CD19, CD20, CD33, and CD123 chimeric antigen receptors, and these mRNA CAR T cells have demonstrated cytotoxicity to their targets [175,176,177,178]. Moreover, the cytotoxicity of mRNA-engineered T cells was controllable [179]. However, what we need to see is that mRNA CAR-Ts are only functional for a certain period of time. Although it is true that serious cytotoxicity does not occur, a single infusion of mRNA CAR-Ts does not result in lasting remission, and multiple transfusions may be required. Therefore, further exploration is needed at this point.

### 4.4. Eliminating CAR-Ts Completely Appropriately

**Figure 3:**
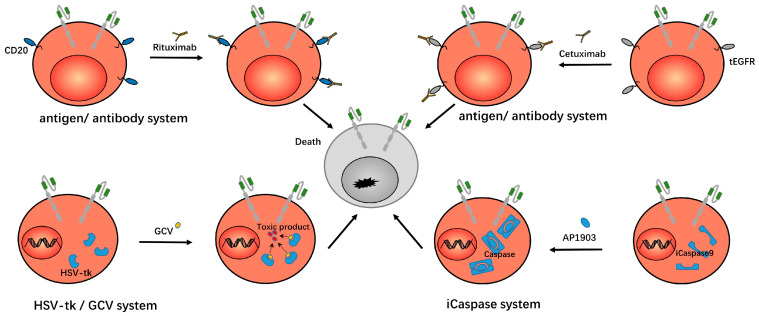
In the antigen/antibody system, researchers expressed CD20/tEGFR on CAR-Ts, which can then be eliminated by rituximab/cetuximab, so as to eliminate the CAR-Ts. In the HSV-tk/GCV system, thymidine kinase (TK) is expressed in CAR-Ts. After the administration of ganciclovir, ganciclovir is metabolized into a toxic product that induces the death of the CAR-Ts. In the iCaspase system, iCaspase9 exists within CAR-Ts. After the administration of AP1903, AP1903 induces the dimerization of iCaspase9 and takes effect, thus inducing the death of CAR-Ts.

Researchers have developed a truncated version of the epidermal growth factor receptor (tEGFR), which is co-expressed with CAR on the surface of T cells. This receptor lacks intracellular tyrosine kinase activity and has no effect on the activities of cells. However, this receptor can bind to cetuximab and induce antibody-dependent, cell-mediated cytotoxicity, thereby eliminating CAR-Ts that cause severe toxicity. In preclinical trials, cetuximab eliminated imported CD19 CAR-Ts in mice, resulting in complete and permanent restoration of normal, functional B cells without tumor recurrence [13,29]. In addition to EGFR, T cells have been studied to express the *RQR8* gene, which encodes cell-surface proteins derived from the CD20 and CD34 epitopes. Therefore, we can use rituximab to eliminate runaway CAR-Ts via antibody-dependent, cell-mediated cytotoxicity (ADCC) [30,31]. Other researchers expressed the *CD20* gene alone on T cells, also using rituximab as a safety switch to control the T cells [32]. This antigen/antibody system is shown in Figure 3.

Another strategy for controlling CAR-Ts that cause severe toxicity is to introduce an inducible killing switch to trigger T cell apoptosis. The caspase-related killing switch is based on modified human caspase9 fused to human FK506 binding protein (FKBP). The dimerizing inducer AP1903 can dimerize caspase protein by cross-linking FKBP, thus inducing cell apoptosis [15,33]. The results showed that AP1903 eliminated 90% of engineered T cells within 30 min [34]. The study in the CD19/CD20/CD33/SLAMF7 CAR-Ts showed that the presence of this suicide gene allowed us to induce the death of CAR-Ts in a dose-dependent manner. That is, the rapid control of the living drug CAR-Ts when it causes uncontrolled toxicity was achieved, and the modified T cells carrying suicide genes can persist and function in the human body for a long time without the addition of dimerizing inducers [39]. This iCaspase system is shown in Figure 3.

The HSV-TK/Ganciclovir system was originally designed to overcome graft versus host disease (GVHD) after allogeneic hematopoietic stem cell transplantation. In this system, the HSV-TK gene was incorporated into T cells. After the ganciclovir was administered, ganciclovir was phosphorylated by thymidine kinase (TK) to produce cytotoxic phosphorylated products which inhibited the activity of DNA polymerase in cells. Or, as a competitive inhibitor of dGTP, this product was incorporated into the synthesized DNA to inhibit DNA synthesis and lead to cell death. [14,40]. This suicide system can be used to control CAR-Ts when they cause severe toxicity. In addition, there is a similar human thymidine kinase (TMPK) variant/azide thymidine (AZT) system. After the TMPK variant was incorporated with T cells, AZT could destroy the mitochondrial intima potential and activate caspase 3 to induce cell apoptosis [180]. This HSV-tk/GCV system is shown in Figure 3.

### 4.5. Reducing the Dose of CAR-Ts to Control Toxicity

According to the conventional cancer treatment reasoning, patients with high tumor burdens should be treated with higher doses of drugs. In adoptive cellular immunotherapy, it also seems appropriate to administer higher cell doses to patients with high tumor burdens. However, high doses of CAR-Ts and tumor burdens increase the risk of severe cytokine release syndrome (sCRS) and neurotoxicity. In a clinical trial, researchers administered lower doses of CAR-Ts to patients with higher tumor burdens. Six out of six patients with a high tumor burden in the control group required ICU care due to severe toxicity compared with only one out of ten patients with a high tumor burden who were treated with the low-dose strategy. In addition, a higher tumor burden implies a greater antigenic stimulation of CAR-Ts which can induce sufficient CAR-Ts to proliferate, thus reducing the dose of CAR-Ts that can exert enough of an effect while reducing toxicity [181].

Another type of dose adjustment for CAR-T therapy is divided administration, i.e., 10% of the total dose on day 1, 30% of the total dose on day 2, and 60% of the total dose on day 3. In clinical trials of adult R/R ALL, this approach has been shown to reduce the severity of CRS and improve safety without compromising the ultimate outcome [182].

## 5. Conclusions

CAR-T therapy in hematologic malignancies has made great progress, but there are still some areas for improvement. For example, life-threatening toxicity, insufficient anti-tumor activity, poor persistence, and the recurrence of tumors occurred in the course of CAR-T therapy. In this paper, we describe various strategies for reducing the toxicity and enhancing the efficacy of CAR-T therapy. For example, T cells can be modified to knock out or overexpress certain genes or inhibit certain effector proteins, thus enhancing the T cells’ activity. Anti-tumor strategies such as BTK inhibitors and immune checkpoint inhibitors were combined to enhance the effects of CAR-Ts. Hinge domains, transmembrane domains, antigen-binding domains, and co-stimulatory domains were adjusted to enhance anti-tumor activity and reduce toxicity during CAR design. Using drugs to control inflammatory cytokines in the presence of life-threatening toxicity to control symptoms directly or controlling CAR-Ts activity with drugs are other possibilities. In the case of life-threatening circumstances during CAR-T therapy, we can even target the elimination of CAR-Ts and terminate CAR-T therapy. In conclusion, to make CAR-T therapy safer and more effective, researchers have developed several strategies. These strategies will contribute to the better application of CAR-T therapy for hematologic malignancies.

## Figures and Tables

**Table 1:** Strategies to enhance the efficacy of CAR-T therapy and reduce its toxicity using gene-editing technologies.

Protein	Effect	Reference
NR4A protein	Associated with CD8+ T cell exhaustion; inhibited the expression of T cells’ effector genes. Knockout of NR4A enhanced CAR-Ts’ anti-tumor function and promoted tumor reduction.	[4,24,25]
TOX protein	Increased inhibitory receptors on T cells’ surfaces; induced exhaustion of T cells. Knockout of TOX enhanced CAR-Ts’ persistence and tumor-killing activity.	[5,26,27,28]
ASS/OTC protein	Overexpressed ASS or OTC enzymes can promote arginine synthesis to drive CAR-T proliferation	[3]
EGFR/CD20 protein	Dysfunctional EGFR or CD20 were expressed on CAR-Ts to facilitate clearance of CAR-Ts using monoclonal antibody drugs when necessary.	[13,29,30,31,32]
caspase9/ FKBP fused protein	FKBP protein was dimerized by the inducer AP1903, which led to the dimerization and activation of caspase9 protein and induced apoptosis of CAR-Ts.	[15,33,34,35,36,37,38,39]
HSV-TK protein	HSV-TK protein was expressed in CAR-Ts. After ganciclovir was administrated, it was phosphorylated by TK protein into cytotoxic products and induced cell death.	[14,40]
Special control system	Special control systems were used to regulate CAR-T activity.	Reference
Tet-on/off system	In this system, doxycycline was used to initiate or terminate T cell proliferation, cytokine production, CAR expression, and cytotoxicity.	[41,42,43,44]
Protease/protease inhibitor system	In this system, the protease cleavage site, protease, and degron series were connected in series after the CAR structure. Using protease inhibitors can degrade the entire sequence, including the CAR structure, and render it useless.	[17]
Antibody-FITC system	In this system, antibody-conjugated FITC molecules and anti-FITC CAR-T were used. The CAR-Ts are only effective after the patient is administered the conjugate molecule.	[11,45,46,47,48]
Special CAR structure		Reference
Tandem CAR-T	Such CAR-Ts have two tandem antigen-recognition domains and target both antigens simultaneously, which can alleviate the problem of antigen-negative tumor recurrence.	[9,49,50,51,52,53,54,55,56]
Dual CAR-T	Such CAR-Ts have two different CAR structures and target both antigens simultaneously, having a similar effect to tandem CAR-T.	[8,57]
Tri-specific CAR-T	Such CAR-Ts targeted three antigens. However, there are two forms, with one CAR-T expressing three different CAR structures. The other CAR-T expresses a tandem CAR structure and another conventional CAR structure.	[10,58]

**Table 2:** Strategies to enhance the efficacy of CAR-T therapy and reduce toxicity using drugs.

Drug	Effect	Reference
Duvelisib	Inhibited the PI3K signal to regulate CAR-Ts’ differentiation; enhanced the activity and function of CAR-Ts.	[7,59]
JQ1	Inhibited BET protein BRD4 to induce CD8+ T cells to exhibit naive T cell characteristics; enhanced CAR-Ts’ persistence and anti-tumor effects.	[6]
Ibrutinib	Enhanced activity of CAR-Ts and promoted the expansion of CAR-Ts by down-regulating PD-1, TIM-3, and LAG-3 on the surfaces of the T cells. Prevented the occurrence of CRS.	[18,60,61,62,63]
Venetoclax	Up-regulated the expression of CD19 in tumor cells so that CAR-Ts exerted a better effect.	[64]
1-methyltrypto phan	Inhibited IDO expression by tumor cells; prevented CAR-Ts from inhibitory metabolites of IDO.	[65,66]
Checkpoint inhibitors	Restored CAR-Ts’ functional activity and persistence; promoted survival of tumor-bearing mice.	[19,20,67,68,69,70,71,72,73,74,75,76,77,78]
Tozzizumab/ Siltuximab	Inhibited IL-6 signaling and reduced the symptoms of cytokine release syndrome.	[21,79,80,81,82,83,84,85,86,87,88]
Anakinra	Inhibited IL-1 receptor in central nervous system, relieved neurotoxicity of patients, and reduced the levels of IL-6 and TNF in serum and cerebrospinal fluid.	[22,89,90]
GM-CSF antibody	Alleviated the toxic effects of CAR-T therapy, such as CRS and neurotoxicity, through neutralizing GM-CSF without inhibiting the anti-tumor activity of CAR-Ts.	[23,91,92,93,94,95,96]
MTP/ANP	Inhibited catecholamines to reduce inflammation response, improved mouse survival without diminishing the expansion and tumor clearance of CAR-Ts.	[97]
Itacitinib/ ruxolitinib	Inhibited JAK-STAT signal pathway, thus inhibiting the functions of various inflammatory cytokines.	[98,99,100]
Dasatinib	Inhibited CD3-based signaling in T cells by inhibiting tyrosine kinase and inducing the temporary inactivation of CAR-Ts; exerted an inhibitory role in the occurrence of severe CRS and neurotoxicity and reduced toxicity. This inhibition is reversible.	[12,101]

## Data Availability

Not applicable.

## Abbreviation

ASS	argininosuccinate synthetase
ALL	acute lymphoblastic leukemia
ANP	atrial natriuretic peptide
BETz	bromodomain and extra-terminal motif
CAR-T	chimeric antigen receptor T cells
CIL	Cytokine-induced killer
CLL	chronic lymphocytic leukemia
CR	complete response
CRS	cytokine release syndrome
GVHD	graft versus host disease
IDO	indoleamine 2,3-dioxygenase
IFN-γ	interferon-γ
LAK	lymphokine-activated killer cell
MDSC	myeloid-derived suppressor cells
MTP	methyltyrosine
NHL	non-Hodgkin lymphoma
NR	no response
ORR	objective response rate
OTC	ornithine transcarbamylase
PD-1	programmed cell death protein 1
scFv	single-chain antibody fragment
TIL	tumor infiltrating lymphocyte

## References

[1] Sterner R.C., Sterner R.M. (2021). CAR-T cell therapy: Current limitations and potential strategies. Blood Cancer J..

[2] Kong Y., Tang L., You Y., Li Q., Zhu X. (2023). Analysis of causes for poor persistence of CAR-T cell therapy in vivo. Front. Immunol..

[3] Fultang L., Booth S., Yogev O., Martins da Costa B., Tubb V., Panetti S., Stavrou V., Scarpa U., Jankevics A., Lloyd G. (2020). Metabolic engineering against the arginine microenvironment enhances CAR-T cell proliferation and therapeutic activity. Blood.

[4] Chen J., López-Moyado I.F., Seo H., Lio C.J., Hempleman L.J., Sekiya T., Yoshimura A., Scott-Browne J.P., Rao A. (2019). NR4A transcription factors limit CAR T cell function in solid tumours. Nature.

[5] Scott A.C., Dündar F., Zumbo P., Chandran S.S., Klebanoff C.A., Shakiba M., Trivedi P., Menocal L., Appleby H., Camara S. (2019). TOX is a critical regulator of tumour-specific T cell differentiation. Nature.

[6] Kagoya Y., Nakatsugawa M., Yamashita Y., Ochi T., Guo T., Anczurowski M., Saso K., Butler M.O., Arrowsmith C.H., Hirano N. (2016). BET bromodomain inhibition enhances T cell persistence and function in adoptive immunotherapy models. J. Clin. Investig..

[7] Zheng W., O’Hear C.E., Alli R., Basham J.H., Abdelsamed H.A., Palmer L.E., Jones L.L., Youngblood B., Geiger T.L. (2018). PI3K orchestration of the in vivo persistence of chimeric antigen receptor-modified T cells. Leukemia.

[8] Ruella M., Barrett D.M., Kenderian S.S., Shestova O., Hofmann T.J., Perazzelli J., Klichinsky M., Aikawa V., Nazimuddin F., Kozlowski M. (2016). Dual CD19 and CD123 targeting prevents antigen-loss relapses after CD19-directed immunotherapies. J. Clin. Investig..

[9] Grada Z., Hegde M., Byrd T., Shaffer D.R., Ghazi A., Brawley V.S., Corder A., Schönfeld K., Koch J., Dotti G. (2013). TanCAR: A Novel Bispecific Chimeric Antigen Receptor for Cancer Immunotherapy. Mol. Ther. Nucleic Acids.

[10] Schneider D., Xiong Y., Wu D., Hu P., Alabanza L., Steimle B., Mahmud H., Anthony-Gonda K., Krueger W., Zhu Z. (2021). Trispecific CD19-CD20-CD22-targeting duoCAR-T cells eliminate antigen-heterogeneous B cell tumors in preclinical models. Sci. Transl. Med..

[11] Ma J.S., Kim J.Y., Kazane S.A., Choi S.H., Yun H.Y., Kim M.S., Rodgers D.T., Pugh H.M., Singer O., Sun S.B. (2016). Versatile strategy for controlling the specificity and activity of engineered T cells. Proc. Natl. Acad. Sci. USA.

[12] Weber E.W., Lynn R.C., Sotillo E., Lattin J., Xu P., Mackall C.L. (2019). Pharmacologic control of CAR-T cell function using dasatinib. Blood Adv..

[13] Paszkiewicz P.J., Fräßle S.P., Srivastava S., Sommermeyer D., Hudecek M., Drexler I., Sadelain M., Liu L., Jensen M.C., Riddell S.R. (2016). Targeted antibody-mediated depletion of murine CD19 CAR T cells permanently reverses B cell aplasia. J. Clin. Investig..

[14] Ciceri F., Bonini C., Marktel S., Zappone E., Servida P., Bernardi M., Pescarollo A., Bondanza A., Peccatori J., Rossini S. (2007). Antitumor effects of HSV-TK-engineered donor lymphocytes after allogeneic stem-cell transplantation. Blood.

[15] Straathof K.C., Pulè M.A., Yotnda P., Dotti G., Vanin E.F., Brenner M.K., Heslop H.E., Spencer D.M., Rooney C.M. (2005). An inducible caspase 9 safety switch for T-cell therapy. Blood.

[16] Griffioen M., van Egmond E.H., Kester M.G., Willemze R., Falkenburg J.H., Heemskerk M.H. (2009). Retroviral transfer of human CD20 as a suicide gene for adoptive T-cell therapy. Haematologica.

[17] Juillerat A., Tkach D., Busser B.W., Temburni S., Valton J., Duclert A., Poirot L., Depil S., Duchateau P. (2019). Modulation of chimeric antigen receptor surface expression by a small molecule switch. BMC Biotechnol..

[18] Fraietta J.A., Beckwith K.A., Patel P.R., Ruella M., Zheng Z., Barrett D.M., Lacey S.F., Melenhorst J.J., McGettigan S.E., Cook D.R. (2016). Ibrutinib enhances chimeric antigen receptor T-cell engraftment and efficacy in leukemia. Blood.

[19] Mumprecht S., Schürch C., Schwaller J., Solenthaler M., Ochsenbein A.F. (2009). Programmed death 1 signaling on chronic myeloid leukemia-specific T cells results in T-cell exhaustion and disease progression. Blood.

[20] Shapiro M., Herishanu Y., Katz B.Z., Dezorella N., Sun C., Kay S., Polliack A., Avivi I., Wiestner A., Perry C. (2017). Lymphocyte activation gene 3: A novel therapeutic target in chronic lymphocytic leukemia. Haematologica.

[21] Le R.Q., Li L., Yuan W., Shord S.S., Nie L., Habtemariam B.A., Przepiorka D., Farrell A.T., Pazdur R. (2018). FDA Approval Summary: Tocilizumab for Treatment of Chimeric Antigen Receptor T Cell-Induced Severe or Life-Threatening Cytokine Release Syndrome. Oncologist.

[22] Giavridis T., van der Stegen S.J.C., Eyquem J., Hamieh M., Piersigilli A., Sadelain M. (2018). CAR T cell-induced cytokine release syndrome is mediated by macrophages and abated by IL-1 blockade. Nat. Med..

[23] Sterner R.M., Sakemura R., Cox M.J., Yang N., Khadka R.H., Forsman C.L., Hansen M.J., Jin F., Ayasoufi K., Hefazi M. (2019). GM-CSF inhibition reduces cytokine release syndrome and neuroinflammation but enhances CAR-T cell function in xenografts. Blood.

[24] Mognol G.P., Spreafico R., Wong V., Scott-Browne J.P., Togher S., Hoffmann A., Hogan P.G., Rao A., Trifari S. (2017). Exhaustion-associated regulatory regions in CD8(+) tumor-infiltrating T cells. Proc. Natl. Acad. Sci. USA.

[25] Liu X., Wang Y., Lu H., Li J., Yan X., Xiao M., Hao J., Alekseev A., Khong H., Chen T. (2019). Genome-wide analysis identifies NR4A1 as a key mediator of T cell dysfunction. Nature.

[26] Seo H., Chen J., González-Avalos E., Samaniego-Castruita D., Das A., Wang Y.H., López-Moyado I.F., Georges R.O., Zhang W., Onodera A. (2019). TOX and TOX2 transcription factors cooperate with NR4A transcription factors to impose CD8(+) T cell exhaustion. Proc. Natl. Acad. Sci. USA.

[27] Khan O., Giles J.R., McDonald S., Manne S., Ngiow S.F., Patel K.P., Werner M.T., Huang A.C., Alexander K.A., Wu J.E. (2019). TOX transcriptionally and epigenetically programs CD8(+) T cell exhaustion. Nature.

[28] Wei J., Long L., Zheng W., Dhungana Y., Lim S.A., Guy C., Wang Y., Wang Y.D., Qian C., Xu B. (2019). Targeting REGNASE-1 programs long-lived effector T cells for cancer therapy. Nature.

[29] Wang X., Chang W.C., Wong C.W., Colcher D., Sherman M., Ostberg J.R., Forman S.J., Riddell S.R., Jensen M.C. (2011). A transgene-encoded cell surface polypeptide for selection, in vivo tracking, and ablation of engineered cells. Blood.

[30] Philip B., Kokalaki E., Mekkaoui L., Thomas S., Straathof K., Flutter B., Marin V., Marafioti T., Chakraverty R., Linch D. (2014). A highly compact epitope-based marker/suicide gene for easier and safer T-cell therapy. Blood.

[31] Vogler I., Newrzela S., Hartmann S., Schneider N., von Laer D., Koehl U., Grez M. (2010). An improved bicistronic CD20/tCD34 vector for efficient purification and in vivo depletion of gene-modified T cells for adoptive immunotherapy. Mol. Ther..

[32] Sommer C., Boldajipour B., Kuo T.C., Bentley T., Sutton J., Chen A., Geng T., Dong H., Galetto R., Valton J. (2019). Preclinical Evaluation of Allogeneic CAR T Cells Targeting BCMA for the Treatment of Multiple Myeloma. Mol. Ther..

[33] Guercio M., Manni S., Boffa I., Caruso S., Di Cecca S., Sinibaldi M., Abbaszadeh Z., Camera A., Ciccone R., Polito V.A. (2021). Inclusion of the Inducible Caspase 9 Suicide Gene in CAR Construct Increases Safety of CAR.CD19 T Cell Therapy in B-Cell Malignancies. Front. Immunol..

[34] Di Stasi A., Tey S.K., Dotti G., Fujita Y., Kennedy-Nasser A., Martinez C., Straathof K., Liu E., Durett A.G., Grilley B. (2011). Inducible apoptosis as a safety switch for adoptive cell therapy. N. Engl. J. Med..

[35] Diaconu I., Ballard B., Zhang M., Chen Y., West J., Dotti G., Savoldo B. (2017). Inducible Caspase-9 Selectively Modulates the Toxicities of CD19-Specific Chimeric Antigen Receptor-Modified T Cells. Mol. Ther..

[36] Budde L.E., Berger C., Lin Y., Wang J., Lin X., Frayo S.E., Brouns S.A., Spencer D.M., Till B.G., Jensen M.C. (2013). Combining a CD20 chimeric antigen receptor and an inducible caspase 9 suicide switch to improve the efficacy and safety of T cell adoptive immunotherapy for lymphoma. PLoS ONE.

[37] Minagawa K., Jamil M.O., Al-Obaidi M., Pereboeva L., Salzman D., Erba H.P., Lamb L.S., Bhatia R., Mineishi S., Di Stasi A. (2016). In Vitro Pre-Clinical Validation of Suicide Gene Modified Anti-CD33 Redirected Chimeric Antigen Receptor T-Cells for Acute Myeloid Leukemia. PLoS ONE.

[38] Amatya C., Pegues M.A., Lam N., Vanasse D., Geldres C., Choi S., Hewitt S.M., Feldman S.A., Kochenderfer J.N. (2021). Development of CAR T Cells Expressing a Suicide Gene Plus a Chimeric Antigen Receptor Targeting Signaling Lymphocytic-Activation Molecule F7. Mol. Ther..

[39] Zhou X., Di Stasi A., Tey S.K., Krance R.A., Martinez C., Leung K.S., Durett A.G., Wu M.F., Liu H., Leen A.M. (2014). Long-term outcome after haploidentical stem cell transplant and infusion of T cells expressing the inducible caspase 9 safety transgene. Blood.

[40] Hsu C., Abad J.D., Morgan R.A. (2013). Characterization of human T lymphocytes engineered to express interleukin-15 and herpes simplex virus-thymidine kinase. J. Surg. Res..

[41] Gu X., He D., Li C., Wang H., Yang G. (2018). Development of Inducible CD19-CAR T Cells with a Tet-On System for Controlled Activity and Enhanced Clinical Safety. Int. J. Mol. Sci..

[42] Sakemura R., Terakura S., Watanabe K., Julamanee J., Takagi E., Miyao K., Koyama D., Goto T., Hanajiri R., Nishida T. (2016). A Tet-On Inducible System for Controlling CD19-Chimeric Antigen Receptor Expression upon Drug Administration. Cancer Immunol. Res..

[43] Drent E., Poels R., Mulders M.J., van de Donk N., Themeli M., Lokhorst H.M., Mutis T. (2018). Feasibility of controlling CD38-CAR T cell activity with a Tet-on inducible CAR design. PLoS ONE.

[44] Mamonkin M., Mukherjee M., Srinivasan M., Sharma S., Gomes-Silva D., Mo F., Krenciute G., Orange J.S., Brenner M.K. (2018). Reversible Transgene Expression Reduces Fratricide and Permits 4-1BB Costimulation of CAR T Cells Directed to T-cell Malignancies. Cancer Immunol. Res..

[45] Lee Y.G., Marks I., Srinivasarao M., Kanduluru A.K., Mahalingam S.M., Liu X., Chu H., Low P.S. (2019). Use of a Single CAR T Cell and Several Bispecific Adapters Facilitates Eradication of Multiple Antigenically Different Solid Tumors. Cancer Res..

[46] Zhang E., Gu J., Xue J., Lin C., Liu C., Li M., Hao J., Setrerrahmane S., Chi X., Qi W. (2018). Accurate control of dual-receptor-engineered T cell activity through a bifunctional anti-angiogenic peptide. J. Hematol. Oncol..

[47] Pellegrino C., Favalli N., Sandholzer M., Volta L., Bassi G., Millul J., Cazzamalli S., Matasci M., Villa A., Myburgh R. (2020). Impact of Ligand Size and Conjugation Chemistry on the Performance of Universal Chimeric Antigen Receptor T-Cells for Tumor Killing. Bioconjug. Chem..

[48] Qi J., Tsuji K., Hymel D., Burke T.R., Hudecek M., Rader C., Peng H. (2020). Chemically Programmable and Switchable CAR-T Therapy. Angew. Chem. Int. Ed. Engl..

[49] Zah E., Lin M.Y., Silva-Benedict A., Jensen M.C., Chen Y.Y. (2016). T Cells Expressing CD19/CD20 Bispecific Chimeric Antigen Receptors Prevent Antigen Escape by Malignant B Cells. Cancer Immunol. Res..

[50] Qin H., Ramakrishna S., Nguyen S., Fountaine T.J., Ponduri A., Stetler-Stevenson M., Yuan C.M., Haso W., Shern J.F., Shah N.N. (2018). Preclinical Development of Bivalent Chimeric Antigen Receptors Targeting Both CD19 and CD22. Mol. Ther. Oncolytics.

[51] Ormhøj M., Scarfò I., Cabral M.L., Bailey S.R., Lorrey S.J., Bouffard A.A., Castano A.P., Larson R.C., Riley L.S., Schmidts A. (2019). Chimeric Antigen Receptor T Cells Targeting CD79b Show Efficacy in Lymphoma with or without Cotargeting CD19. Clin. Cancer Res..

[52] Scarfò I., Ormhøj M., Frigault M.J., Castano A.P., Lorrey S., Bouffard A.A., van Scoyk A., Rodig S.J., Shay A.J., Aster J.C. (2018). Anti-CD37 chimeric antigen receptor T cells are active against B- and T-cell lymphomas. Blood.

[53] Spiegel J.Y., Patel S., Muffly L., Hossain N.M., Oak J., Baird J.H., Frank M.J., Shiraz P., Sahaf B., Craig J. (2021). CAR T cells with dual targeting of CD19 and CD22 in adult patients with recurrent or refractory B cell malignancies: A phase 1 trial. Nat. Med..

[54] Hu Y., Zhou Y., Zhang M., Ge W., Li Y., Yang L., Wei G., Han L., Wang H., Yu S. (2021). CRISPR/Cas9-Engineered Universal CD19/CD22 Dual-Targeted CAR-T Cell Therapy for Relapsed/Refractory B-cell Acute Lymphoblastic Leukemia. Clin. Cancer Res..

[55] Tong C., Zhang Y., Liu Y., Ji X., Zhang W., Guo Y., Han X., Ti D., Dai H., Wang C. (2020). Optimized tandem CD19/CD20 CAR-engineered T cells in refractory/relapsed B-cell lymphoma. Blood.

[56] Schneider D., Xiong Y., Wu D., Nölle V., Schmitz S., Haso W., Kaiser A., Dropulic B., Orentas R.J. (2017). A tandem CD19/CD20 CAR lentiviral vector drives on-target and off-target antigen modulation in leukemia cell lines. J. Immunother. Cancer.

[57] (2021). Dual CD19/CD22 CAR T Cells Show Feasibility in Pediatric/Young Adult B-ALL. Cancer Discov..

[58] Fousek K., Watanabe J., Joseph S.K., George A., An X., Byrd T.T., Morris J.S., Luong A., Martínez-Paniagua M.A., Sanber K. (2021). CAR T-cells that target acute B-lineage leukemia irrespective of CD19 expression. Leukemia.

[59] Funk C.R., Wang S., Chen K.Z., Waller A., Sharma A., Edgar C.L., Gupta V.A., Chandrakasan S., Zoine J.T., Fedanov A. (2022). PI3Kδ/γ inhibition promotes human CART cell epigenetic and metabolic reprogramming to enhance antitumor cytotoxicity. Blood.

[60] Gauthier J., Hirayama A.V., Purushe J., Hay K.A., Lymp J., Li D.H., Yeung C.C.S., Sheih A., Pender B.S., Hawkins R.M. (2020). Feasibility and efficacy of CD19-targeted CAR T cells with concurrent ibrutinib for CLL after ibrutinib failure. Blood.

[61] Long M., Beckwith K., Do P., Mundy B.L., Gordon A., Lehman A.M., Maddocks K.J., Cheney C., Jones J.A., Flynn J.M. (2017). Ibrutinib treatment improves T cell number and function in CLL patients. J. Clin. Investig..

[62] Fan F., Yoo H.J., Stock S., Wang L., Liu Y., Schubert M.L., Wang S., Neuber B., Hückelhoven-Krauss A., Gern U. (2021). Ibrutinib for improved chimeric antigen receptor T-cell production for chronic lymphocytic leukemia patients. Int. J. Cancer.

[63] Ruella M., Kenderian S.S., Shestova O., Klichinsky M., Melenhorst J.J., Wasik M.A., Lacey S.F., June C.H., Gill S. (2017). Kinase inhibitor ibrutinib to prevent cytokine-release syndrome after anti-CD19 chimeric antigen receptor T cells for B-cell neoplasms. Leukemia.

[64] Yang M., Wang L., Ni M., Neuber B., Wang S., Gong W., Sauer T., Sellner L., Schubert M.L., Hückelhoven-Krauss A. (2020). Pre-sensitization of Malignant B Cells Through Venetoclax Significantly Improves the Cytotoxic Efficacy of CD19.CAR-T Cells. Front. Immunol..

[65] Ricciuti B., Leonardi G.C., Puccetti P., Fallarino F., Bianconi V., Sahebkar A., Baglivo S., Chiari R., Pirro M. (2019). Targeting indoleamine-2,3-dioxygenase in cancer: Scientific rationale and clinical evidence. Pharmacol. Ther..

[66] Ninomiya S., Narala N., Huye L., Yagyu S., Savoldo B., Dotti G., Heslop H.E., Brenner M.K., Rooney C.M., Ramos C.A. (2015). Tumor indoleamine 2,3-dioxygenase (IDO) inhibits CD19-CAR T cells and is downregulated by lymphodepleting drugs. Blood.

[67] McLane L.M., Abdel-Hakeem M.S., Wherry E.J. (2019). CD8 T Cell Exhaustion During Chronic Viral Infection and Cancer. Annu. Rev. Immunol..

[68] Speiser D.E., Ho P.C., Verdeil G. (2016). Regulatory circuits of T cell function in cancer. Nat. Rev. Immunol..

[69] Kenderian S.S., Ruella M., Shestova O., Klichinsky M., Kim M.Y., Porter D.L., June C.H., Gill S.I. (2015). Identification of PD1 and TIM3 As Checkpoints That Limit Chimeric Antigen Receptor T Cell Efficacy in Leukemia. Blood.

[70] Deng Q., Han G., Puebla-Osorio N., Ma M.C.J., Strati P., Chasen B., Dai E., Dang M., Jain N., Yang H. (2020). Characteristics of anti-CD19 CAR T cell infusion products associated with efficacy and toxicity in patients with large B cell lymphomas. Nat. Med..

[71] Finney O.C., Brakke H.M., Rawlings-Rhea S., Hicks R., Doolittle D., Lopez M., Futrell R.B., Orentas R.J., Li D., Gardner R.A. (2019). CD19 CAR T cell product and disease attributes predict leukemia remission durability. J. Clin. Investig..

[72] Li A.M., Hucks G.E., Dinofia A.M., Seif A.E., Teachey D.T., Baniewicz D., Callahan C., Fasano C., McBride B., Gonzalez V. (2018). Checkpoint Inhibitors Augment CD19-Directed Chimeric Antigen Receptor (CAR) T Cell Therapy in Relapsed B-Cell Acute Lymphoblastic Leukemia. Blood.

[73] Toffalori C., Zito L., Gambacorta V., Riba M., Oliveira G., Bucci G., Barcella M., Spinelli O., Greco R., Crucitti L. (2019). Immune signature drives leukemia escape and relapse after hematopoietic cell transplantation. Nat. Med..

[74] Zhou J.E., Yu J., Wang Y., Wang H., Wang J., Wang Y., Yu L., Yan Z. (2021). ShRNA-mediated silencing of PD-1 augments the efficacy of chimeric antigen receptor T cells on subcutaneous prostate and leukemia xenograft. Biomed Pharm..

[75] Ren J., Liu X., Fang C., Jiang S., June C.H., Zhao Y. (2017). Multiplex Genome Editing to Generate Universal CAR T Cells Resistant to PD1 Inhibition. Clin. Cancer Res..

[76] Hu B., Zou Y., Zhang L., Tang J., Niedermann G., Firat E., Huang X., Zhu X. (2019). Nucleofection with Plasmid DNA for CRISPR/Cas9-Mediated Inactivation of Programmed Cell Death Protein 1 in CD133-Specific CAR T Cells. Hum. Gene Ther..

[77] Yin Y., Boesteanu A.C., Binder Z.A., Xu C., Reid R.A., Rodriguez J.L., Cook D.R., Thokala R., Blouch K., McGettigan-Croce B. (2018). Checkpoint Blockade Reverses Anergy in IL-13Rα2 Humanized scFv-Based CAR T Cells to Treat Murine and Canine Gliomas. Mol. Ther. Oncolytics.

[78] Cao Y., Lu W., Sun R., Jin X., Cheng L., He X., Wang L., Yuan T., Lyu C., Zhao M. (2019). Anti-CD19 Chimeric Antigen Receptor T Cells in Combination With Nivolumab Are Safe and Effective Against Relapsed/Refractory B-Cell Non-hodgkin Lymphoma. Front. Oncol..

[79] Venkiteshwaran A. (2009). Tocilizumab. MAbs.

[80] Maude S.L., Frey N., Shaw P.A., Aplenc R., Barrett D.M., Bunin N.J., Chew A., Gonzalez V.E., Zheng Z., Lacey S.F. (2014). Chimeric antigen receptor T cells for sustained remissions in leukemia. N. Engl. J. Med..

[81] Maude S.L., Barrett D., Teachey D.T., Grupp S.A. (2014). Managing cytokine release syndrome associated with novel T cell-engaging therapies. Cancer J..

[82] Grupp S.A., Kalos M., Barrett D., Aplenc R., Porter D.L., Rheingold S.R., Teachey D.T., Chew A., Hauck B., Wright J.F. (2013). Chimeric antigen receptor-modified T cells for acute lymphoid leukemia. N. Engl. J. Med..

[83] Gardner R.A., Ceppi F., Rivers J., Annesley C., Summers C., Taraseviciute A., Gust J., Leger K.J., Tarlock K., Cooper T.M. (2019). Preemptive mitigation of CD19 CAR T-cell cytokine release syndrome without attenuation of antileukemic efficacy. Blood.

[84] Nishimoto N., Terao K., Mima T., Nakahara H., Takagi N., Kakehi T. (2008). Mechanisms and pathologic significances in increase in serum interleukin-6 (IL-6) and soluble IL-6 receptor after administration of an anti-IL-6 receptor antibody, tocilizumab, in patients with rheumatoid arthritis and Castleman disease. Blood.

[85] Kurzrock R., Voorhees P.M., Casper C., Furman R.R., Fayad L., Lonial S., Borghaei H., Jagannath S., Sokol L., Usmani S.Z. (2013). A phase I, open-label study of siltuximab, an anti-IL-6 monoclonal antibody, in patients with B-cell non-Hodgkin lymphoma, multiple myeloma, or Castleman disease. Clin. Cancer Res..

[86] van Rhee F., Fayad L., Voorhees P., Furman R., Lonial S., Borghaei H., Sokol L., Crawford J., Cornfeld M., Qi M. (2010). Siltuximab, a novel anti-interleukin-6 monoclonal antibody, for Castleman’s disease. J. Clin. Oncol..

[87] Lipe B.C., Renaud T. (2022). Siltuximab as a primary treatment for cytokine release syndrome in a patient receiving a bispecific antibody in a clinical trial setting. J. Oncol. Pharm. Pract..

[88] Tan A.H.J., Vinanica N., Campana D. (2020). Chimeric antigen receptor-T cells with cytokine neutralizing capacity. Blood Adv..

[89] Wehrli M., Gallagher K., Chen Y.B., Leick M.B., McAfee S.L., El-Jawahri A.R., DeFilipp Z., Horick N., O’Donnell P., Spitzer T. (2022). Single-center experience using anakinra for steroid-refractory immune effector cell-associated neurotoxicity syndrome (ICANS). J. Immunother. Cancer.

[90] Sandler R.D., Carter S., Kaur H., Francis S., Tattersall R.S., Snowden J.A. (2020). Haemophagocytic lymphohistiocytosis (HLH) following allogeneic haematopoietic stem cell transplantation (HSCT)-time to reappraise with modern diagnostic and treatment strategies?. Bone Marrow Transpl..

[91] Sachdeva M., Duchateau P., Depil S., Poirot L., Valton J. (2019). Granulocyte-macrophage colony-stimulating factor inactivation in CAR T-cells prevents monocyte-dependent release of key cytokine release syndrome mediators. J. Biol. Chem..

[92] Gust J., Hay K.A., Hanafi L.A., Li D., Myerson D., Gonzalez-Cuyar L.F., Yeung C., Liles W.C., Wurfel M., Lopez J.A. (2017). Endothelial Activation and Blood-Brain Barrier Disruption in Neurotoxicity after Adoptive Immunotherapy with CD19 CAR-T Cells. Cancer Discov..

[93] Sterner R.M., Kenderian S.S. (2020). Myeloid cell and cytokine interactions with chimeric antigen receptor-T-cell therapy: Implication for future therapies. Curr. Opin. Hematol..

[94] Neelapu S.S., Locke F.L., Bartlett N.L., Lekakis L.J., Miklos D.B., Jacobson C.A., Braunschweig I., Oluwole O.O., Siddiqi T., Lin Y. (2017). Axicabtagene Ciloleucel CAR T-Cell Therapy in Refractory Large B-Cell Lymphoma. N. Engl. J. Med..

[95] Sterner R.M., Cox M.J., Sakemura R., Kenderian S.S. (2019). Using CRISPR/Cas9 to Knock Out GM-CSF in CAR-T Cells. J. Vis. Exp..

[96] Burga R.A., Thorn M., Point G.R., Guha P., Nguyen C.T., Licata L.A., DeMatteo R.P., Ayala A., Joseph Espat N., Junghans R.P. (2015). Liver myeloid-derived suppressor cells expand in response to liver metastases in mice and inhibit the anti-tumor efficacy of anti-CEA CAR-T. Cancer Immunol. Immunother..

[97] Staedtke V., Bai R.Y., Kim K., Darvas M., Davila M.L., Riggins G.J., Rothman P.B., Papadopoulos N., Kinzler K.W., Vogelstein B. (2018). Disruption of a self-amplifying catecholamine loop reduces cytokine release syndrome. Nature.

[98] Huarte E., O’Connor R.S., Peel M.T., Nunez-Cruz S., Leferovich J., Juvekar A., Yang Y.O., Truong L., Huang T., Naim A. (2020). Itacitinib (INCB039110), a JAK1 Inhibitor, Reduces Cytokines Associated with Cytokine Release Syndrome Induced by CAR T-cell Therapy. Clin. Cancer Res..

[99] Li S., Wang X., Yuan Z., Liu L., Luo L., Li Y., Wu K., Liu J., Yang C., Li Z. (2021). Eradication of T-ALL Cells by CD7-targeted Universal CAR-T Cells and Initial Test of Ruxolitinib-based CRS Management. Clin. Cancer Res..

[100] Zi F.M., Ye L.L., Zheng J.F., Cheng J., Wang Q.M. (2021). Using JAK inhibitor to treat cytokine release syndrome developed after chimeric antigen receptor T cell therapy for patients with refractory acute lymphoblastic leukemia: A case report. Medicine.

[101] Mestermann K., Giavridis T., Weber J., Rydzek J., Frenz S., Nerreter T., Mades A., Sadelain M., Einsele H., Hudecek M. (2019). The tyrosine kinase inhibitor dasatinib acts as a pharmacologic on/off switch for CAR T cells. Sci. Transl. Med..

[102] Zhang X., Jin X., Sun R., Zhang M., Lu W., Zhao M. (2022). Gene knockout in cellular immunotherapy: Application and limitations. Cancer Lett..

[103] Pavlovic K., Tristán-Manzano M., Maldonado-Pérez N., Cortijo-Gutierrez M., Sánchez-Hernández S., Justicia-Lirio P., Carmona M.D., Herrera C., Martin F., Benabdellah K. (2020). Using Gene Editing Approaches to Fine-Tune the Immune System. Front. Immunol..

[104] Huang N., Huang Z., Gao M., Luo Z., Zhou F., Liu L., Xiao Q., Wang X., Feng W. (2018). Induction of apoptosis in imatinib sensitive and resistant chronic myeloid leukemia cells by efficient disruption of bcr-abl oncogene with zinc finger nucleases. J. Exp. Clin. Cancer Res..

[105] Conway A., Mendel M., Kim K., McGovern K., Boyko A., Zhang L., Miller J.C., DeKelver R.C., Paschon D.E., Mui B.L. (2019). Non-viral Delivery of Zinc Finger Nuclease mRNA Enables Highly Efficient In Vivo Genome Editing of Multiple Therapeutic Gene Targets. Mol. Ther..

[106] Qasim W., Zhan H., Samarasinghe S., Adams S., Amrolia P., Stafford S., Butler K., Rivat C., Wright G., Somana K. (2017). Molecular remission of infant B-ALL after infusion of universal TALEN gene-edited CAR T cells. Sci. Transl. Med..

[107] Liu X., Zhang Y., Cheng C., Cheng A.W., Zhang X., Li N., Xia C., Wei X., Liu X., Wang H. (2017). CRISPR-Cas9-mediated multiplex gene editing in CAR-T cells. Cell Res..

[108] Seki A., Rutz S. (2018). Optimized RNP transfection for highly efficient CRISPR/Cas9-mediated gene knockout in primary T cells. J. Exp. Med..

[109] Dai X., Park J.J., Du Y., Na Z., Lam S.Z., Chow R.D., Renauer P.A., Gu J., Xin S., Chu Z. (2023). Massively parallel knock-in engineering of human T cells. Nat. Biotechnol..

[110] Rabilloud T., Potier D., Pankaew S., Nozais M., Loosveld M., Payet-Bornet D. (2021). Single-cell profiling identifies pre-existing CD19-negative subclones in a B-ALL patient with CD19-negative relapse after CAR-T therapy. Nat. Commun..

[111] Gardner R., Wu D., Cherian S., Fang M., Hanafi L.A., Finney O., Smithers H., Jensen M.C., Riddell S.R., Maloney D.G. (2016). Acquisition of a CD19-negative myeloid phenotype allows immune escape of MLL-rearranged B-ALL from CD19 CAR-T-cell therapy. Blood.

[112] Vacaflores A., Freedman S.N., Chapman N.M., Houtman J.C. (2017). Pretreatment of activated human CD8 T cells with IL-12 leads to enhanced TCR-induced signaling and cytokine production. Mol. Immunol..

[113] Vacaflores A., Chapman N.M., Harty J.T., Richer M.J., Houtman J.C. (2016). Exposure of Human CD4 T Cells to IL-12 Results in Enhanced TCR-Induced Cytokine Production, Altered TCR Signaling, and Increased Oxidative Metabolism. PLoS ONE.

[114] Choi J.N., Sun E.G., Cho S.H. (2019). IL-12 Enhances Immune Response by Modulation of Myeloid Derived Suppressor Cells in Tumor Microenvironment. Chonnam Med. J..

[115] Kueberuwa G., Kalaitsidou M., Cheadle E., Hawkins R.E., Gilham D.E. (2018). CD19 CAR T Cells Expressing IL-12 Eradicate Lymphoma in Fully Lymphoreplete Mice through Induction of Host Immunity. Mol. Ther. Oncolytics.

[116] Avanzi M.P., Yeku O., Li X., Wijewarnasuriya D.P., van Leeuwen D.G., Cheung K., Park H., Purdon T.J., Daniyan A.F., Spitzer M.H. (2018). Engineered Tumor-Targeted T Cells Mediate Enhanced Anti-Tumor Efficacy Both Directly and through Activation of the Endogenous Immune System. Cell Rep..

[117] Hoyos V., Savoldo B., Quintarelli C., Mahendravada A., Zhang M., Vera J., Heslop H.E., Rooney C.M., Brenner M.K., Dotti G. (2010). Engineering CD19-specific T lymphocytes with interleukin-15 and a suicide gene to enhance their anti-lymphoma/leukemia effects and safety. Leukemia.

[118] Kasakovski D., Xu L., Li Y. (2018). T cell senescence and CAR-T cell exhaustion in hematological malignancies. J. Hematol. Oncol..

[119] Riches J.C., Davies J.K., McClanahan F., Fatah R., Iqbal S., Agrawal S., Ramsay A.G., Gribben J.G. (2013). T cells from CLL patients exhibit features of T-cell exhaustion but retain capacity for cytokine production. Blood.

[120] Hoffmann J.M., Schubert M.L., Wang L., Hückelhoven A., Sellner L., Stock S., Schmitt A., Kleist C., Gern U., Loskog A. (2017). Differences in Expansion Potential of Naive Chimeric Antigen Receptor T Cells from Healthy Donors and Untreated Chronic Lymphocytic Leukemia Patients. Front. Immunol..

[121] Fraietta J.A., Lacey S.F., Orlando E.J., Pruteanu-Malinici I., Gohil M., Lundh S., Boesteanu A.C., Wang Y., O’Connor R.S., Hwang W.T. (2018). Determinants of response and resistance to CD19 chimeric antigen receptor (CAR) T cell therapy of chronic lymphocytic leukemia. Nat. Med..

[122] Nishimura T., Kaneko S., Kawana-Tachikawa A., Tajima Y., Goto H., Zhu D., Nakayama-Hosoya K., Iriguchi S., Uemura Y., Shimizu T. (2013). Generation of rejuvenated antigen-specific T cells by reprogramming to pluripotency and redifferentiation. Cell Stem Cell.

[123] Crompton J.G., Clever D., Vizcardo R., Rao M., Restifo N.P. (2014). Reprogramming antitumor immunity. Trends. Immunol..

[124] Jin X., Lu W., Zhang M., Xiong X., Sun R., Wei Y., He X., Zhao M. (2021). Infection Temperature Affects the Phenotype and Function of Chimeric Antigen Receptor T Cells Produced via Lentiviral Technology. Front. Immunol..

[125] Kaartinen T., Luostarinen A., Maliniemi P., Keto J., Arvas M., Belt H., Koponen J., Mäkinen P.I., Loskog A., Mustjoki S. (2017). Low interleukin-2 concentration favors generation of early memory T cells over effector phenotypes during chimeric antigen receptor T-cell expansion. Cytotherapy.

[126] Gattinoni L., Finkelstein S.E., Klebanoff C.A., Antony P.A., Palmer D.C., Spiess P.J., Hwang L.N., Yu Z., Wrzesinski C., Heimann D.M. (2005). Removal of homeostatic cytokine sinks by lymphodepletion enhances the efficacy of adoptively transferred tumor-specific CD8+ T cells. J. Exp. Med..

[127] Xu Y., Zhang M., Ramos C.A., Durett A., Liu E., Dakhova O., Liu H., Creighton C.J., Gee A.P., Heslop H.E. (2014). Closely related T-memory stem cells correlate with in vivo expansion of CAR.CD19-T cells and are preserved by IL-7 and IL-15. Blood.

[128] Hurton L.V., Singh H., Najjar A.M., Switzer K.C., Mi T., Maiti S., Olivares S., Rabinovich B., Huls H., Forget M.A. (2016). Tethered IL-15 augments antitumor activity and promotes a stem-cell memory subset in tumor-specific T cells. Proc. Natl. Acad. Sci. USA.

[129] Alizadeh D., Wong R.A., Yang X., Wang D., Pecoraro J.R., Kuo C.F., Aguilar B., Qi Y., Ann D.K., Starr R. (2019). IL15 Enhances CAR-T Cell Antitumor Activity by Reducing mTORC1 Activity and Preserving Their Stem Cell Memory Phenotype. Cancer Immunol. Res..

[130] Tormo A., Khodayarian F., Cui Y., Al-Chami E., Kanjarawi R., Noé B., Wang H., Rafei M. (2017). Interleukin-21 promotes thymopoiesis recovery following hematopoietic stem cell transplantation. J. Hematol. Oncol..

[131] Al-Chami E., Tormo A., Pasquin S., Kanjarawi R., Ziouani S., Rafei M. (2016). Interleukin-21 administration to aged mice rejuvenates their peripheral T-cell pool by triggering de novo thymopoiesis. Aging Cell.

[132] Singh H., Figliola M.J., Dawson M.J., Huls H., Olivares S., Switzer K., Mi T., Maiti S., Kebriaei P., Lee D.A. (2011). Reprogramming CD19-specific T cells with IL-21 signaling can improve adoptive immunotherapy of B-lineage malignancies. Cancer Res..

[133] Baird J.H., Frank M.J., Craig J., Patel S., Spiegel J.Y., Sahaf B., Oak J.S., Younes S.F., Ozawa M.G., Yang E. (2021). CD22-directed CAR T-cell therapy induces complete remissions in CD19-directed CAR-refractory large B-cell lymphoma. Blood.

[134] Wang N., Hu X., Cao W., Li C., Xiao Y., Cao Y., Gu C., Zhang S., Chen L., Cheng J. (2020). Efficacy and safety of CAR19/22 T-cell cocktail therapy in patients with refractory/relapsed B-cell malignancies. Blood.

[135] Zhang Y., Li S., Wang Y., Lu Y., Xu Y., Rao Q., Wang H., Xing H., Tian Z., Tang K. (2022). A novel and efficient CD22 CAR-T therapy induced a robust antitumor effect in relapsed/refractory leukemia patients when combined with CD19 CAR-T treatment as a sequential therapy. Exp. Hematol. Oncol..

[136] Yan N., Wang N., Wang G., Huang L., Li C., Wang D., Wang J., Huang L., Meng F., Wei J. (2022). CAR19/22 T cell cocktail therapy for B-ALL relapsed after allogeneic hematopoietic stem cell transplantation. Cytotherapy.

[137] Yan L., Qu S., Shang J., Shi X., Kang L., Xu N., Zhu M., Zhou J., Jin S., Yao W. (2021). Sequential CD19 and BCMA-specific CAR T-cell treatment elicits sustained remission of relapsed and/or refractory myeloma. Cancer Med..

[138] Meng Y., Deng B., Rong L., Li C., Song W., Ling Z., Xu J., Duan J., Wang Z., Chang A.H. (2021). Short-Interval Sequential CAR-T Cell Infusion May Enhance Prior CAR-T Cell Expansion to Augment Anti-Lymphoma Response in B-NHL. Front. Oncol..

[139] Yan L.Z., Shang J.J., Kang L.Q., Shi X.L., Zhou J., Jin S., Yao W.Q., Yao Y., Chen G.H., Zhu Z.L. (2017). Combined Infusion of CD19 and Bcma-Specific Chimeric Antigen Receptor T Cells for RRMM: Initial Safety and Efficacy Report from a Clinical Pilot Study. Blood.

[140] Liu Y., Deng B., Hu B., Zhang W., Zhu Q., Liu Y., Wang S., Zhang P., Yang Y., Yang J. (2022). Sequential different B-cell antigen-targeted CAR T-cell therapy for pediatric refractory/relapsed Burkitt lymphoma. Blood Adv..

[141] Du J., Zhang Y. (2020). Sequential anti-CD19, 22, and 20 autologous chimeric antigen receptor T-cell (CAR-T) treatments of a child with relapsed refractory Burkitt lymphoma: A case report and literature review. J. Cancer Res. Clin. Oncol..

[142] Sommermeyer D., Hill T., Shamah S.M., Salter A.I., Chen Y., Mohler K.M., Riddell S.R. (2017). Fully human CD19-specific chimeric antigen receptors for T-cell therapy. Leukemia.

[143] Maus M.V., Haas A.R., Beatty G.L., Albelda S.M., Levine B.L., Liu X., Zhao Y., Kalos M., June C.H. (2013). T cells expressing chimeric antigen receptors can cause anaphylaxis in humans. Cancer Immunol. Res..

[144] Cao J., Wang G., Cheng H., Wei C., Qi K., Sang W., Zhenyu L., Shi M., Li H., Qiao J. (2018). Potent anti-leukemia activities of humanized CD19-targeted Chimeric antigen receptor T (CAR-T) cells in patients with relapsed/refractory acute lymphoblastic leukemia. Am. J. Hematol..

[145] Brudno J.N., Lam N., Vanasse D., Shen Y.W., Rose J.J., Rossi J., Xue A., Bot A., Scholler N., Mikkilineni L. (2020). Safety and feasibility of anti-CD19 CAR T cells with fully human binding domains in patients with B-cell lymphoma. Nat. Med..

[146] Brudno J.N., Hartman S.D., Pittaluga S., Stroncek D., Lam N., Kanakry J.A., Pavletic S.Z., Mikkilineni L., Bagheri M., Roschewski M.J. (2018). Clinical anti-lymphoma activity and toxicity of T cells expressing a novel anti-CD19 chimeric antigen receptor with fully-human variable regions. J. Clin. Oncol..

[147] Almåsbak H., Walseng E., Kristian A., Myhre M.R., Suso E.M., Munthe L.A., Andersen J.T., Wang M.Y., Kvalheim G., Gaudernack G. (2015). Inclusion of an IgG1-Fc spacer abrogates efficacy of CD19 CAR T cells in a xenograft mouse model. Gene Ther..

[148] Haso W., Lee D.W., Shah N.N., Stetler-Stevenson M., Yuan C.M., Pastan I.H., Dimitrov D.S., Morgan R.A., FitzGerald D.J., Barrett D.M. (2013). Anti-CD22-chimeric antigen receptors targeting B-cell precursor acute lymphoblastic leukemia. Blood.

[149] Schäfer D., Henze J., Pfeifer R., Schleicher A., Brauner J., Mockel-Tenbrinck N., Barth C., Gudert D., Al Rawashdeh W., Johnston I.C.D. (2020). A Novel Siglec-4 Derived Spacer Improves the Functionality of CAR T Cells Against Membrane-Proximal Epitopes. Front. Immunol..

[150] Hombach A., Hombach A.A., Abken H. (2010). Adoptive immunotherapy with genetically engineered T cells: Modification of the IgG1 Fc ‘spacer’ domain in the extracellular moiety of chimeric antigen receptors avoids ‘off-target’ activation and unintended initiation of an innate immune response. Gene Ther..

[151] Hudecek M., Sommermeyer D., Kosasih P.L., Silva-Benedict A., Liu L., Rader C., Jensen M.C., Riddell S.R. (2015). The nonsignaling extracellular spacer domain of chimeric antigen receptors is decisive for in vivo antitumor activity. Cancer Immunol. Res..

[152] Gonzalez-Garcia P., Muñoz-Miranda J.P., Fernandez-Cisnal R., Olvera L., Moares N., Gabucio A., Fernandez-Ponce C., Garcia-Cozar F. (2023). Specific Activation of T Cells by an ACE2-Based CAR-Like Receptor upon Recognition of SARS-CoV-2 Spike Protein. Int. J. Mol. Sci..

[153] Majzner R.G., Rietberg S.P., Sotillo E., Dong R., Vachharajani V.T., Labanieh L., Myklebust J.H., Kadapakkam M., Weber E.W., Tousley A.M. (2020). Tuning the Antigen Density Requirement for CAR T-cell Activity. Cancer Discov..

[154] Alabanza L., Pegues M., Geldres C., Shi V., Wiltzius J.J.W., Sievers S.A., Yang S., Kochenderfer J.N. (2017). Function of Novel Anti-CD19 Chimeric Antigen Receptors with Human Variable Regions Is Affected by Hinge and Transmembrane Domains. Mol. Ther..

[155] Ying Z., Huang X.F., Xiang X., Liu Y., Kang X., Song Y., Guo X., Liu H., Ding N., Zhang T. (2019). A safe and potent anti-CD19 CAR T cell therapy. Nat. Med..

[156] Freyer C.W., Porter D.L. (2020). Cytokine release syndrome and neurotoxicity following CAR T-cell therapy for hematologic malignancies. J. Allergy Clin. Immunol..

[157] Kawalekar O.U., O’Connor R.S., Fraietta J.A., Guo L., McGettigan S.E., Posey A.D., Patel P.R., Guedan S., Scholler J., Keith B. (2016). Distinct Signaling of Coreceptors Regulates Specific Metabolism Pathways and Impacts Memory Development in CAR T Cells. Immunity.

[158] Salter A.I., Ivey R.G., Kennedy J.J., Voillet V., Rajan A., Alderman E.J., Voytovich U.J., Lin C., Sommermeyer D., Liu L. (2018). Phosphoproteomic analysis of chimeric antigen receptor signaling reveals kinetic and quantitative differences that affect cell function. Sci. Signal..

[159] Milone M.C., Fish J.D., Carpenito C., Carroll R.G., Binder G.K., Teachey D., Samanta M., Lakhal M., Gloss B., Danet-Desnoyers G. (2009). Chimeric receptors containing CD137 signal transduction domains mediate enhanced survival of T cells and increased antileukemic efficacy in vivo. Mol. Ther..

[160] van der Stegen S.J., Hamieh M., Sadelain M. (2015). The pharmacology of second-generation chimeric antigen receptors. Nat. Rev. Drug Discov..

[161] Long A.H., Haso W.M., Shern J.F., Wanhainen K.M., Murgai M., Ingaramo M., Smith J.P., Walker A.J., Kohler M.E., Venkateshwara V.R. (2015). 4-1BB costimulation ameliorates T cell exhaustion induced by tonic signaling of chimeric antigen receptors. Nat. Med..

[162] Zhao X., Yang J., Zhang X., Lu X.A., Xiong M., Zhang J., Zhou X., Qi F., He T., Ding Y. (2020). Efficacy and Safety of CD28- or 4-1BB-Based CD19 CAR-T Cells in B Cell Acute Lymphoblastic Leukemia. Mol. Ther. Oncolytics.

[163] Schmidts A., Wehrli M., Maus M.V. (2021). Toward Better Understanding and Management of CAR-T Cell-Associated Toxicity. Annu. Rev. Med..

[164] Tedesco V.E.t., Mohan C. (2021). Biomarkers for Predicting Cytokine Release Syndrome following CD19-Targeted CAR T Cell Therapy. J. Immunol..

[165] Norelli M., Camisa B., Barbiera G., Falcone L., Purevdorj A., Genua M., Sanvito F., Ponzoni M., Doglioni C., Cristofori P. (2018). Monocyte-derived IL-1 and IL-6 are differentially required for cytokine-release syndrome and neurotoxicity due to CAR T cells. Nat. Med..

[166] Teachey D.T., Lacey S.F., Shaw P.A., Melenhorst J.J., Maude S.L., Frey N., Pequignot E., Gonzalez V.E., Chen F., Finklestein J. (2016). Identification of Predictive Biomarkers for Cytokine Release Syndrome after Chimeric Antigen Receptor T-cell Therapy for Acute Lymphoblastic Leukemia. Cancer Discov..

[167] Santomasso B.D., Park J.H., Salloum D., Riviere I., Flynn J., Mead E., Halton E., Wang X., Senechal B., Purdon T. (2018). Clinical and Biological Correlates of Neurotoxicity Associated with CAR T-cell Therapy in Patients with B-cell Acute Lymphoblastic Leukemia. Cancer Discov..

[168] Garbers C., Heink S., Korn T., Rose-John S. (2018). Interleukin-6: Designing specific therapeutics for a complex cytokine. Nat. Rev. Drug Discov..

[169] Jones S.A., Jenkins B.J. (2018). Recent insights into targeting the IL-6 cytokine family in inflammatory diseases and cancer. Nat. Rev. Immunol..

[170] Jiang Z., Liao R., Lv J., Li S., Zheng D., Qin L., Wu D., Chen S., Long Y., Wu Q. (2021). IL-6 trans-signaling promotes the expansion and anti-tumor activity of CAR T cells. Leukemia.

[171] Jatiani S.S., Aleman A., Madduri D., Chari A., Cho H.J., Richard S., Richter J., Brody J., Jagannath S., Parekh S. (2020). Myeloma CAR-T CRS Management With IL-1R Antagonist Anakinra. Clin. Lymphoma Myeloma Leuk..

[172] Zhang L., Wang S., Xu J., Zhang R., Zhu H., Wu Y., Zhu L., Li J., Chen L. (2021). Etanercept as a new therapeutic option for cytokine release syndrome following chimeric antigen receptor T cell therapy. Exp. Hematol. Oncol..

[173] Zhou W., Chen W., Wan X., Luo C., Du X., Li X., Chen Q., Gao R., Zhang X., Xie M. (2021). Benefits of Chimeric Antigen Receptor T-Cell Therapy for B-Cell Lymphoma. Front. Genet..

[174] Miliotou A.N., Papadopoulou L.C. (2020). In Vitro-Transcribed (IVT)-mRNA CAR Therapy Development. Methods Mol. Biol..

[175] Almåsbak H., Rian E., Hoel H.J., Pulè M., Wälchli S., Kvalheim G., Gaudernack G., Rasmussen A.M. (2011). Transiently redirected T cells for adoptive transfer. Cytotherapy.

[176] Panjwani M.K., Smith J.B., Schutsky K., Gnanandarajah J., O’Connor C.M., Powell D.J., Mason N.J. (2016). Feasibility and Safety of RNA-transfected CD20-specific Chimeric Antigen Receptor T Cells in Dogs with Spontaneous B Cell Lymphoma. Mol. Ther..

[177] Kenderian S.S., Ruella M., Shestova O., Klichinsky M., Aikawa V., Morrissette J.J., Scholler J., Song D., Porter D.L., Carroll M. (2015). CD33-specific chimeric antigen receptor T cells exhibit potent preclinical activity against human acute myeloid leukemia. Leukemia.

[178] Tasian S.K., Kenderian S.S., Shen F., Ruella M., Shestova O., Kozlowski M., Li Y., Schrank-Hacker A., Morrissette J.J.D., Carroll M. (2017). Optimized depletion of chimeric antigen receptor T cells in murine xenograft models of human acute myeloid leukemia. Blood.

[179] Barrett D.M., Zhao Y., Liu X., Jiang S., Carpenito C., Kalos M., Carroll R.G., June C.H., Grupp S.A. (2011). Treatment of advanced leukemia in mice with mRNA engineered T cells. Hum. Gene Ther..

[180] Sato T., Neschadim A., Konrad M., Fowler D.H., Lavie A., Medin J.A. (2007). Engineered human tmpk/AZT as a novel enzyme/prodrug axis for suicide gene therapy. Mol. Ther..

[181] Turtle C.J., Hanafi L.A., Berger C., Gooley T.A., Cherian S., Hudecek M., Sommermeyer D., Melville K., Pender B., Budiarto T.M. (2016). CD19 CAR-T cells of defined CD4+:CD8+ composition in adult B cell ALL patients. J. Clin. Investig..

[182] Frey N.V., Shaw P.A., Hexner E.O., Pequignot E., Gill S., Luger S.M., Mangan J.K., Loren A.W., Perl A.E., Maude S.L. (2020). Optimizing Chimeric Antigen Receptor T-Cell Therapy for Adults With Acute Lymphoblastic Leukemia. J. Clin. Oncol..

